# Active Travel Mode and Incident Dementia and Brain Structure

**DOI:** 10.1001/jamanetworkopen.2025.14316

**Published:** 2025-06-09

**Authors:** Cunpeng Hou, Yaqi Zhang, Feiyang Zhao, Yanling Lv, Mengyun Luo, Chensheng Pan, Ding Ding, Liangkai Chen

**Affiliations:** 1 Department of Nutrition and Food Hygiene, Hubei Key Laboratory of Food Nutrition and Safety, School of Public Health, Tongji Medical College, Huazhong University of Science and Technology, Wuhan, China; 2 Ministry of Education Key Lab of Environment and Health, School of Public Health, Tongji Medical College, Huazhong University of Science and Technology, Wuhan, China; 3 Prevention Research Collaboration, Sydney School of Public Health, The University of Sydney, New South Wales, Australia; 4 Charles Perkins Centre, The University of Sydney, New South Wales, Australia; 5 Department of Neurology, Tongji Hospital, Tongji Medical College, Huazhong University of Science and Technology, Wuhan, China

## Abstract

**Importance:**

Active travel modes, such as walking and cycling, are feasible and readily embraced forms of physical activity, but their association with dementia risk and brain structure remains unclear.

**Objectives:**

To investigate the long-term association between travel modes and dementia risk and brain structural metrics and to evaluate whether genetic predisposition could modify the association between travel modes and dementia risk.

**Design, Setting, and Participants:**

This prospective cohort study included data collected from the UK Biobank from March 13, 2006, to October 1, 2010. Data were analyzed from March to October 2024.

**Exposures:**

Travel modes assessed using the question “In the last 4 weeks, which forms of transport have you used most often to get about (not including any journeys to and from work)?” and categorized into 4 groups: nonactive, walking, mixed-walking, and cycling and mixed-cycling mode.

**Main Outcomes and Measures:**

The incidence of all-cause dementia (including young-onset dementia [YOD] and late-onset dementia [LOD]) was the primary outcome, and dementia subtypes, such as Alzheimer disease (AD) and brain structure, were the secondary outcomes; all were identified through hospital records and death registers. Brain structure was measured by a magnetic resonance imaging scan. Travel mode and risk of incident dementia were assessed using Cox proportional hazards regression models with hazard ratios and 95% CIs.

**Results:**

The study encompassed 479 723 participants (mean [SD] age, 56.5 [8.1] years; 260 730 females [54.4%]), including 271 690 in the YOD analyses and 334 939 in the LOD analyses. Over a median follow-up of 13.1 years (IQR, 12.8-13.5 years), 8845 cases of dementia (1.8%) and 3956 cases of AD (0.8%) were recorded. Compared with a nonactive travel mode, multivariable-adjusted hazard ratios for cycling and mixed-cycling were 0.81 (95% CI, 0.73-0.91) for all-cause dementia, 0.78 (95% CI, 0.66-0.92) for AD, 0.60 (95% CI, 0.38-0.95) for YOD, and 0.83 (95% CI, 0.75-0.93) for LOD. A significant interaction between travel mode and genetic susceptibility in the all-cause dementia analysis (*P* = .02 for interaction) and the LOD analysis (*P* = .04 for interaction) was observed; specifically, for the cycling and mixed-cycling groups, the risks of all-cause dementia and LOD were lower among those without apolipoprotein E ε4 (*APOE* ε4) carrier status (all-cause dementia: hazard ratio [HR], 0.74 [95% CI, 0.63-0.87]; LOD: HR, 0.75 [95% CI, 0.63-0.89]) compared with those with *APOE* ε4 carrier status (all-cause dementia: HR, 0.88 [95% CI, 0.76-1.02]; LOD: HR, 0.91 [95% CI, 0.78-1.05]). The cycling and mixed-cycling mode was significantly associated with a higher hippocampal volume (β, 0.05 [95% CI, 0.02-0.08]). Genetic risk significantly modified the association with all-cause dementia (*P* = .02 for interaction) and LOD (*P* = .04 for interaction). Specifically, for the cycling and mixed-cycling groups, the risk of all-cause dementia was lower among those without *APOE* ε4 (HR, 0.74 [95% CI, 0.63-0.87]) compared with those with *APOE* ε4 (HR, 0.88 [95% CI, 0.76-1.02]). Similarly, the risk of LOD was lower among those without *APOE* ε4 (HR, 0.75 [95% CI, 0.63-0.89]) compared with those with *APOE* ε4 (HR, 0.91 [95% CI, 0.78-1.05]).

**Conclusions and Relevance:**

The findings of this cohort study suggest an association between active travel mode and incident dementia and brain structure. The cycling and mixed-cycling mode was associated with a reduced risk of all-cause dementia, including YOD, LOD, and AD, as well as an increased hippocampal volume, suggesting a promising approach for maintaining brain health.

## Key Points

**Question:**

Is active travel mode independently associated with a lower risk of incident dementia?

**Findings:**

In this cohort study of 479 723 participants with over 13 years of follow-up, an active travel mode, particularly cycling and mixed-cycling, was associated with a lower incidence of all-cause dementia, including young-onset and late-onset dementia. Apolipoprotein E ε4 carrier status significantly modified the association between travel modes and dementia risk, particularly for all-cause dementia and late-onset dementia.

**Meaning:**

The findings of this study suggest that promoting active travel modes, particularly cycling, may be associated with better brain health and lower dementia risk.

## Teaser

This cohort study investigates the association of active travel modes, such as walking and cycling, with dementia risk and brain structure.

## Introduction

Dementia has emerged as a leading contributor to dependence and disability among older adults, with the global number of cases projected to rise from 55 million in 2019 to 139 million by 2050.^1^ Young-onset dementia (YOD), characterized by the onset of symptoms before age 65 years,^2,3^ affects an estimated 3.9 million people worldwide.^4^ Although less prevalent than late-onset dementia (LOD), YOD entails a considerably higher care burden^5^ and mortality.^6^ The 2024 *Lancet* Commission identified 14 modifiable risk factors responsible for approximately 45% of dementia cases and emphasized the contribution of physical activity (PA) during middle age as a preventative measure.^7^ However, risk assessments for YOD, particularly in relation to factors with broad public health relevance, remain underexplored.^8^

While the health benefits of PA are well established, an estimated 1.4 billion adults worldwide do not meet the World Health Organization’s recommended levels of PA for maintaining health.^9^ Active travel mode, which involves walking or cycling for transportation (excluding work commutes), typically requires moderate-intensity exertion,^10^ rendering it a feasible and readily embraced form of PA. Different travel modes may not only reflect the level of PA but also encompass various health-related factors, such as environmental exposure, attentional engagement, and spatial navigation abilities.^11^ Although population-based evidence on the health effects of active travel remains scarce, systematic reviews have consistently highlighted its association with improved health outcomes, including a lower risk of diabetes.^12^ However, few studies have investigated the association between travel mode and brain health (eg, dementia risk and brain structural changes).

Both genetic and environmental factors contribute to the development of dementia. The health benefits of active travel may vary across populations with different genetic predispositions. In this context, we aimed to investigate the long-term association between various travel modes and the risk of all-cause dementia (including YOD and LOD), dementia subtypes such as Alzheimer disease (AD), and brain structural metrics within a large prospective UK cohort. Additionally, we sought to evaluate whether genetic predisposition could modify the association between travel modes and dementia risk.

## Methods

### Study Population

Between March 13, 2006, and October 1, 2010, the UK Biobank recruited over 500 000 participants, aged 40 to 69 years, from 22 assessment centers in England, Scotland, and Wales.^13^ In this large prospective cohort study, we collected a wealth of information on sociodemographic characteristics, lifestyles, physical examinations, and medical conditions. The study was approved by the North West Multi-centre Research Ethics Committee, and written informed consent was provided before participation. The study followed the Strengthening the Reporting of Observational Studies in Epidemiology (STROBE) reporting guideline.

We excluded individuals who were diagnosed with dementia at baseline or within 2 years of follow-up (n = 3291), individuals who were unable to walk (n = 1866), and individuals lacking travel mode data (n = 6165). In the genetic interaction analysis, additional exclusions were made for individuals with incomplete genetic data or discordance between genetic and self-reported sex (n = 11 572). Furthermore, individuals younger than 60 years and diagnosed with dementia before age 65 years were included in the YOD analyses, while those diagnosed with dementia at age 65 years or older were included in the LOD analyses (eFigure 1 in Supplement 1).

### Travel Mode Assessment

Travel mode data were obtained from touchscreen questionnaires. Baseline assessments used responses to the question “In the last 4 weeks, which forms of transport have you used most often to get about (not including any journeys to and from work)?” Respondents could select 1 or more of the following options: car or motor vehicle, walking, public transporter, and cycle. The exposure variable was categorized into the following 4 groups: nonactive (car or motor vehicle or public transporter), walking, mixed-walking (combination of nonactive and walking), and cycling and mixed-cycling (cycling combined with other modes) modes.

### Outcomes

The primary outcome of interest was the incidence of all-cause dementia (YOD and LOD), and the secondary outcomes included dementia subtypes (ie, AD and brain structure). Individuals with dementia were identified using the *International Classification of Diseases, Ninth Revision* (*ICD-9*) and the *International Statistical Classification of Diseases, Tenth Revision* (*ICD-10*) codes, as recommended by the UK Biobank algorithm^14^ (eTable 1 in Supplement 1). Diagnosis information was sourced from the inpatient electronic health records and death registries through linkage with the Hospital Episode Statistics (for England), the Scottish Morbidity Records (for Scotland), or the Patient Episode Database (for Wales).

### Brain Magnetic Resonance Imaging Scans

Since 2014, the UK Biobank has initiated multimodal imaging studies aimed at acquiring magnetic resonance imaging (MRI) data for the brain, heart, and abdomen of 100 000 individuals.^15^ Brain MRI data were made available to approved researchers as image-derived phenotypes, and we included 96 cortical and 14 subcortical regional gray matter volume (GMV) regions in this study, which were normalized for head size.^16^ Details regarding data acquisition protocols and preprocessing are available elsewhere.^15,17^ The specific field identification numbers used in this study are described in eTable 2 in Supplement 1.

### Genetic Susceptibility to Dementia

Methodologies used for genotyping, imputation, and quality control within the UK Biobank have been reported previously.^18^ The apolipoprotein E (*APOE*) gene stands out as the strongest determinant of dementia risk,^19^ with genotype determination predicated on 2 single-nucleotide polymorphisms, denoted as rs429358 and rs7412. Participants were categorized as those with *APOE* ε4 or those without *APOE* ε4 accordingly.

### Covariates

Information on age, sex, and race and ethnicity was obtained using a self-administered touchscreen survey.^13^ The race and ethnicity categories included Asian or Asian British, Black or Black British, Chinese, White, more than 1 race or ethnicity, other race or ethnicity (specific categories not provided in the UK Biobank), and unknown race or ethnicity. Race and ethnicity were included in the study because the UK Biobank included multiple racial and ethnic groups, and race and ethnicity are important confounding variables. Socioeconomic status was evaluated using the Townsend Deprivation Index (in which scores range from −6.26 to 11.00, with higher scores indicating greater deprivation), derived from national census data pertaining to the participant’s postal code area.^20^ Smoking status was categorized as current, former, or never, while alcohol consumption was classified based on frequency (never or special occasions only, 1 to 3 times per month, once or twice per week, 3 or 4 times per week, daily or almost daily). Body mass index (BMI) was calculated as weight in kilograms divided by height in meters squared and categorized as 18.5 or less, 18.6 to 24.9, 25.0 to 29.9, 30 or more, or unknown. PA was assessed using the International Physical Activity Questionnaire (IPAQ), which includes low, moderate, and high categories of physical activity, with higher scores indicating vigorous-intensity participation. History of hypertension, cardiovascular disease, dyslipidemia, diabetes, and depression were identified using the *ICD-9* and *ICD-10* illness codes and self-reported fields as described previously.^21^ The status of long-standing illness, disability, or infirmity was collected as part of each participant’s health and medical history at baseline recruitment. We also considered assessment centers (22 categories), employment status, *APOE* ε4 carrier status, and baseline cognitive function (ie, reaction time measured by mean [SD] time to correctly identify matches). Covariate definitions are provided (eTable 3 in Supplement 1).

### Statistical Analysis

Data were analyzed from March to October 2024. The statistical analysis plan was preregistered on Open Science Framework.^22^ Associations between travel mode and the risk of incident dementia were assessed using Cox proportional hazards regression models, with hazard ratios (HRs) and 95% CIs calculated. Follow-up time was denoted from baseline to the date of dementia diagnosis, death, or censorship (in England: October 31, 2022; in Scotland: July 31, 2021; and in Wales: February 28, 2018), whichever came first. The Schoenfeld residual method was adopted to check the proportional hazards assumption. Participants with nonactive travel modes were considered as the reference group, and HRs for walking, mixed-walking, and cycling and mixed-cycling modes were estimated. Three models were used for analyses. Model 1 (minimize model) was adjusted for age, sex, race and ethnicity, educational level, the Townsend Deprivation Index, employment status, and assessment centers. Model 2 included additional adjustments for smoking status, alcohol consumption, PA (IPAQ), and BMI. Model 3 was further adjusted for history of hypertension; dyslipidemia; diabetes; cardiovascular disease; depression; long-standing illness, disability, or infirmity; cognitive function (ie, reaction time); and genetic variables (*APOE* ε4 carrier status). Missing covariate values were coded as a missing indicator category for categorical variables and with median values for continuous variables. The missing proportion of covariates is shown in eTable 4 in Supplement 1.

Linear mixed-effects models were applied to investigate the association of travel mode with regional GMVs.^23,24^ Travel mode was treated as a fixed effect and assessment center as a random effect, with each GMV region analyzed as a separate outcome. Brain association analyses were corrected using the Benjamini-Hochberg false discovery rate method due to the many brain regions. We extracted the standardized β coefficients and converted them to Cohen *d* according to previous methodologies.^25^ Genetic interaction analyses were conducted by introducing a multiplicative interaction term (travel mode × *APOE*-related risk) in the fully adjusted model, followed by subgroup analysis.

We performed the following 8 secondary analyses: (1) stratified analyses by sex, the Townsend Deprivation Index, and *APOE* ε4 status; (2) sensitivity analyses conducted without adjusting for the IPAQ as a covariate, as PA might act as a mediating variable; (3) sensitivity analyses by repeating the main analysis using complete cases only; (4) sensitivity analyses without the aged less than 60 years’ inclusion criterion for YOD; (5) further categorization of nonactive travel modes into public transporter, car or motor vehicle, and mixed nonactive groups (ie, public transporter and car or motor vehicle); (6) validation of brain volume associations using generalized linear models of MRI metrics; (7) investigation of reverse causality by adjusting commuting mode in the association of travel mode with incident dementia in employed participants; and (8) robustness checks using Fine and Gray models to account for competing risk of death. All statistical analyses were conducted using SAS, version 9.4 (SAS Institute Inc); R, version 4.3.2 (R Project for Statistical Computing); and Pycharm, version 2024.2.4 (JetBrains), with statistical significance set at a 2-tailed *P* < .05.

## Results

The study included 479 723 participants (mean [SD] age, 56.5 [8.1] years; 260 730 females [54.4%] and 218 993 males [45.6%]). Among them, 271 690 middle-aged adults (mean [SD] age, 50.7 [5.6] years; 150 990 females [55.6%] and 120 700 males [44.4%]) were included in the YOD analysis, and 334 939 older adults (mean [SD] age, 60.9 [4.9] years; 181 521 females [54.2%] and 153 418 males [45.8%]) were in the LOD analysis. In terms of race and ethnicity, 9109 participants (1.9%) were Asian or Asian British, 7525 (1.6%) were Black or Black British, 1477 (0.3%) were Chinese, 452 924 (94.4%) were White, 2807 (0.6%) were of more than 1 race or ethnicity, 4305 (0.9%) were of other race or ethnicity, and 1576 (0.3%) were of unknown race or ethnicity, and over 85.0% of individuals were of European ancestry. Compared with individuals with nonactive travel modes, those who chose active travel modes (walking, mixed-walking, and cycling and mixed-cycling) were more likely to be female, nonsmokers, more educated, engaged in more PA, with a lower BMI, and with fewer chronic diseases (Table 1 and eTables 5 and 6 in Supplement 1). Furthermore, the cycling and mixed-cycling groups were more likely to be male and had a healthier lifestyle and physical condition compared with the others groups.

**Table 1.** Baseline Characteristics of Participants and Associations of Travel Mode With Incident All-Cause Dementia and AD^a^

Characteristic	Travel mode			
	Nonactive	Walking	Mixed-walking	Cycling and mixed-cycling
No. of participants	235 508 (49.1)	32 836 (6.8)	177 635 (37.0)	33 744 (7.0)
Age, mean (SD), y	56.4 (8.0)	56.0 (8.0)	57.1 (8.1)	54.5 (8.3)
Sex				
Female	127 162 (54.0)	18 374 (56.0)	102 750 (57.8)	12 444 (36.9)
Male	108 346 (46.0)	14 462 (44.0)	74 885 (42.2)	21 300 (63.1)
Race and ethnicity				
Asian or Asian British	5452 (2.3)	724 (2.2)	2729 (1.5)	204 (0.6)
Black or Black British	4437 (1.9)	554 (1.7)	2311 (1.3)	223 (0.7)
Chinese	833 (0.4)	156 (0.5)	415 (0.2)	73 (0.2)
White	220 342 (93.6)	30 632 (93.3)	169 282 (95.3)	32 668 (96.8)
More than 1	1433 (0.6)	252 (0.8)	901 (0.5)	221 (0.7)
Other^b^	2345 (1.0)	361 (1.1)	1384 (0.8)	215 (0.6)
Unknown	666 (0.3)	157 (0.5)	613 (0.3)	140 (0.4)
Educational level				
College or university	66 832 (28.4)	10 330 (31.5)	62 523 (35.2)	16 430 (48.7)
Vocational	30 726 (13.0)	4123 (12.6)	18 421 (10.4)	3124 (9.3)
Upper secondary	24 036 (10.2)	3185 (9.7)	22 463 (12.6)	3876 (11.5)
Lower secondary	64 186 (27.3)	8221 (25.0)	48 123 (27.1)	7088 (21.0)
Other	46 893 (19.9)	6492 (19.8)	24 648 (13.9)	3010 (8.9)
Unknown	2835 (1.2)	485 (1.5)	1457 (0.8)	216 (0.6)
Townsend Deprivation Index, median (IQR)^c^	−2.3 (−3.7 to 0.3)	−1.1 (−3.1 to 1.8)	−2.2 (−3.7 to 0.4)	−2.1 (−3.6 to 0.5)
Smoking status				
Never	125 220 (53.2)	17 528 (53.4)	100 762 (56.7)	18 817 (55.8)
Former	82 048 (34.8)	11 074 (33.7)	60 540 (34.1)	12 005 (35.6)
Current	27 241 (11.6)	4072 (12.4)	15 783 (8.9)	2837 (8.4)
Alcohol consumption				
Daily or almost daily	46 120 (19.6)	6474 (19.7)	36 956 (20.8)	8276 (24.5)
3 or 4 Times per wk	52 127 (22.1)	7065 (21.5)	42 631 (24.0)	9500 (28.2)
Once or twice per wk	61 463 (26.1)	8354 (25.4)	45 622 (25.7)	8532 (25.3)
1-3 Times per mo	26 546 (11.3)	3591 (10.9)	20 084 (11.3)	3225 (9.6)
Never or special occasions only	49 020 (20.8)	7300 (22.2)	32 210 (18.1)	4191 (12.4)
IPAQ activity group				
Low	48 109 (20.4)	2517 (7.7)	20 173 (11.4)	1635 (4.8)
Moderate	72 812 (30.9)	11 338 (34.5)	65 735 (37.0)	9016 (26.7)
High	64 969 (27.6)	13 526 (41.2)	59 972 (33.8)	19 002 (56.3)
Unknown	49 618 (21.1)	5455 (16.6)	31 755 (17.9)	4091 (12.1)
Body mass index, mean (SD)^d^	28.0 (5.0)	26.5 (4.4)	27.1 (4.6)	25.9 (3.8)
Employment status				
Employed	141 799 (60.2)	18 651 (56.8)	93 318 (52.5)	22 563 (66.9)
Unemployed	93 709 (39.8)	14 185 (43.2)	84 317 (47.5)	11 181 (33.1)
Comorbidity history				
Diabetes	16 597 (7.0)	1707 (5.2)	9892 (5.6)	1034 (3.1)
Dyslipidemia	132 346 (56.2)	15 999 (48.7)	93 032 (52.4)	14 785 (43.8)
Hypertension	133 547 (56.7)	18 037 (54.9)	100 016 (56.3)	15 598 (46.2)
Cardiovascular disease	18 518 (7.9)	2048 (6.2)	11 824 (6.7)	1455 (4.3)
Depression	27 107 (11.5)	3668 (11.2)	19 982 (11.2)	3254 (9.6)
Long-standing illness, disability, or infirmity	79 005 (33.5)	9435 (28.7)	53 974 (30.4)	7960 (23.6)
Reaction time, mean (SD), ms	559.8 (119.0)	562.6 (121.4)	560.0 (114.7)	538.6 (106.7)
With *APOE* ε4 carrier status	66 489 (28.2)	9356 (28.5)	50 808 (28.6)	9735 (28.8)

Abbreviations: AD, Alzheimer disease; *APOE*, apolipoprotein E; IPAQ, International Physical Activity Questionnaire (scoring includes low, moderate, and high categories of physical activity, with higher scores indicating vigorous-intensity participation).

^a^ Data are presented as the No. (%) of participants unless otherwise indicated.

^b^ Groups not provided in the UK Biobank.

^c^ Scores range from −6.26 to 11.00, with higher scores indicating greater deprivation.

^d^ Calculated as weight in kilograms divided by height in meters squared.

During a median follow-up of 13.1 years (IQR, 12.8-13.5 years), we identified 8845 participants with incident dementia (1.8%), including 528 with YOD (0.2%), 8276 with LOD (2.5%), and 3956 with incident AD (0.8%) (eTable 7 in Supplement 1). We combined the cycling model and the mixed-cycling mode because there were only 3 participants with YOD in the cycling-only mode.

**Figure 1.**
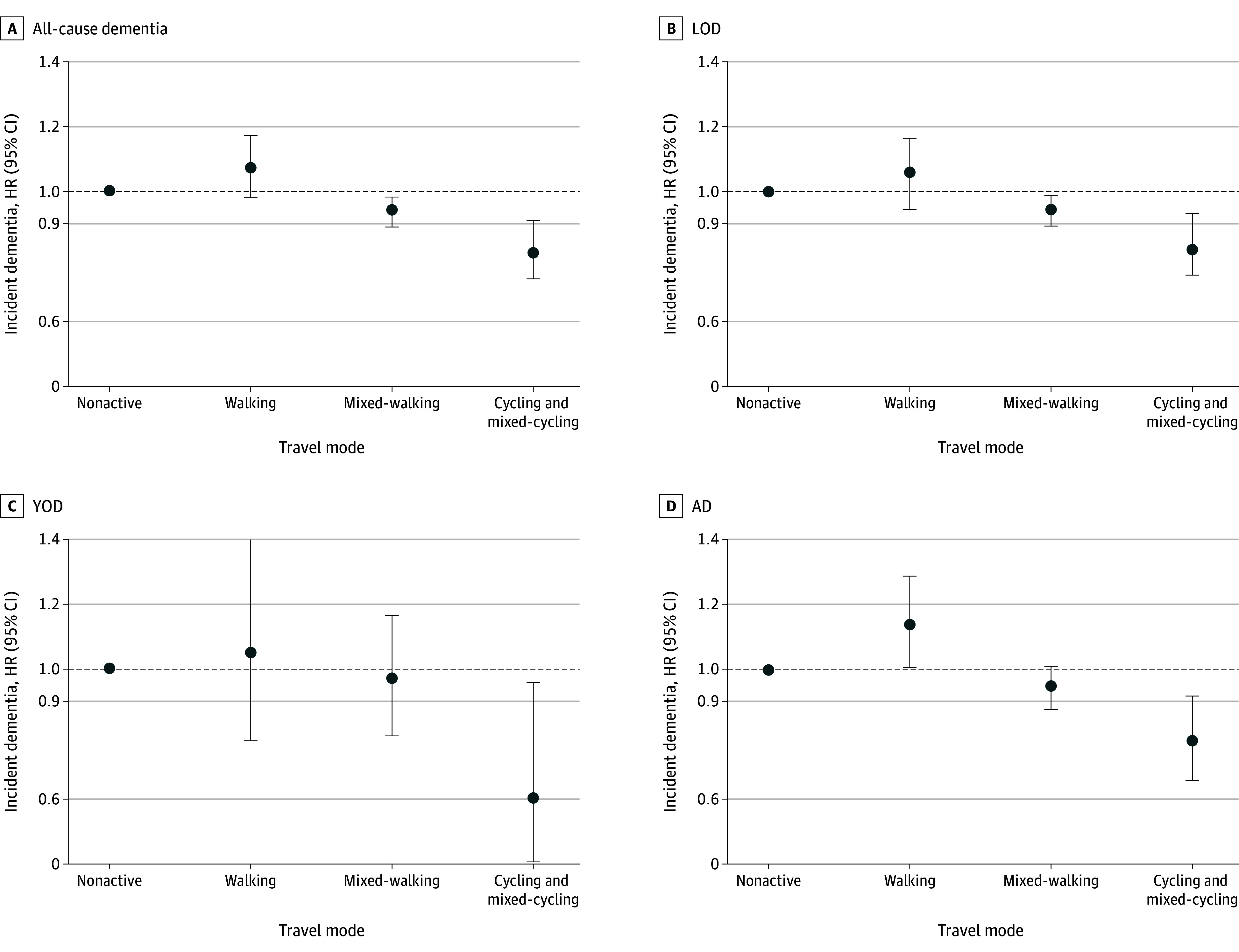
Association Between Travel Mode and Incident All-Cause Dementia, Late-Onset Dementia (LOD), Young-Onset Dementia (YOD), and Alzheimer Disease (AD) Adjusted for age; sex; race and ethnicity; educational level; Townsend Deprivation Index; assessment centers; alcohol consumption; smoking status; body mass index; International Physical Activity Questionnaire activity group; employment status; diabetes; dyslipidemia; hypertension; cardiovascular disease; depression; long-standing illness, disability, or infirmity; cognitive function (ie, reaction time); and genetic variables (apolipoprotein E ε4 carrier status). HR indicates hazard ratio; error bars, 95% CIs.

Compared with individuals who chose a nonactive travel mode, those who chose mixed-walking (HR, 0.94 [95% CI, 0.89-0.98]) and cycling and mixed-cycling (HR, 0.81 [95% CI, 0.73-0.91]) modes showed a significantly decreased risk of all-cause dementia after adjustment for all covariates (Figure 1 and Table 2). For LOD, mixed-walking (HR, 0.94 [95% CI, 0.89-0.98) and cycling and mixed-cycling (HR, 0.83 [95% CI, 0.75-0.93]) modes were also associated with a lower risk (eTable 8 in Supplement 1). Furthermore, cycling and mixed-cycling showed a significantly decreased YOD risk (HR, 0.60 [95% CI, 0.38-0.95]) after full covariate adjustment (eTable 9 in Supplement 1). Interestingly, walking was associated with an increased risk of AD (HR, 1.14 [95% CI, 1.01-1.29]), while cycling and mixed-cycling remained protective (HR, 0.78 [95% CI, 0.66-0.92]) (eFigure 2 and eTable 10 in Supplement 1).

**Table 2.** Association Between Travel Mode and Incident All-Cause Dementia

Characteristic	Travel mode			
	Nonactive	Walking	Mixed-walking	Cycling and mixed-cycling
No. of participants/person-years	4532/3 079 442	628/430 638	3296/2 328 850	389/450 156
Incidence per 100 000 person-years	147.2	145.8	141.5	86.4
Model, hazard ratio (95% CI)				
1^a^	1 [Reference]	1.01 (0.93-1.10)	0.89 (0.85-0.93)	0.72 (0.65-0.80)
2^b^	1 [Reference]	1.04 (0.95-1.13)	0.92 (0.87-0.96)	0.76 (0.69-0.85)
3^c^	1 [Reference]	1.07 (0.98-1.17)	0.94 (0.89-0.98)	0.81 (0.73-0.91)

^a^ Adjusted for age, sex, race and ethnicity, educational level, the Townsend Deprivation Index, employment status, and assessment centers.

^b^ Includes model 1 and alcohol consumption, smoking status, body mass index, and the International Physical Activity Questionnaire activity group.

^c^ Includes model 2 and diabetes; dyslipidemia; hypertension; cardiovascular disease; depression; long-standing illness, disability, or infirmity; cognitive function (ie, reaction time); and genetic variables (apolipoprotein E ε4 carrier status).

Further categorization of nonactive travel modes revealed that both car or motor vehicle (HR, 0.78 [95% CI, 0.72-0.85]) and combined car or motor vehicle with public transporter (HR, 0.81 [95% CI, 0.73-0.91]) were associated with a significantly lower risk of all-cause dementia compared with public transporter alone (Table 3). Although the point estimates suggest a lower risk of dementia for the car or motor vehicle–only group compared with the combined car or motor vehicle with public transporter group, the overlapping 95% CIs indicate no statistically significant difference between these groups. Similar findings were observed for LOD and AD (eTables 11 and 12 in Supplement 1). Notably, only the car or motor vehicle mode was associated with reduced YOD risk (HR, 0.61 [95% CI, 0.44-0.83]) compared with only public transporter in model 2, but this association lost significance upon full covariate adjustment (eFigure 3 and eTable 13 in Supplement 1).

**Table 3.** Association Between Nonactive Travel Mode and Incident All-Cause Dementia

Characteristic	Nonactive travel mode		
	Only public transporter	Only car or motor vehicle	Public transporter and car or motor vehicle
No. of participants/person-years	1022/382 701	3005/2 464 786	505/231 955
Incidence per 100 000 person-years	267.0	121.9	217.7
Model, hazard ratio (95% CI)			
1^a^	1 [Reference]	0.74 (0.69-0.81)	0.74 (0.69-0.81)
2^b^	1 [Reference]	0.75 (0.69-0.81)	0.79 (0.71-0.88)
3^c^	1 [Reference]	0.78 (0.72-0.85)	0.81 (0.73-0.91)

^a^ Adjusted for age, sex, race and ethnicity, educational level, the Townsend Deprivation Index, employment status, and assessment centers.

^b^ Includes model 1 and alcohol consumption, smoking status, body mass index, and the International Physical Activity Questionnaire activity group.

^c^ Includes model 2 and diabetes; dyslipidemia; hypertension; cardiovascular disease; depression; long-standing illness, disability, or infirmity; cognitive function (ie, reaction time); and genetic variables (apolipoprotein E ε4 carrier status).

**Figure 2.**
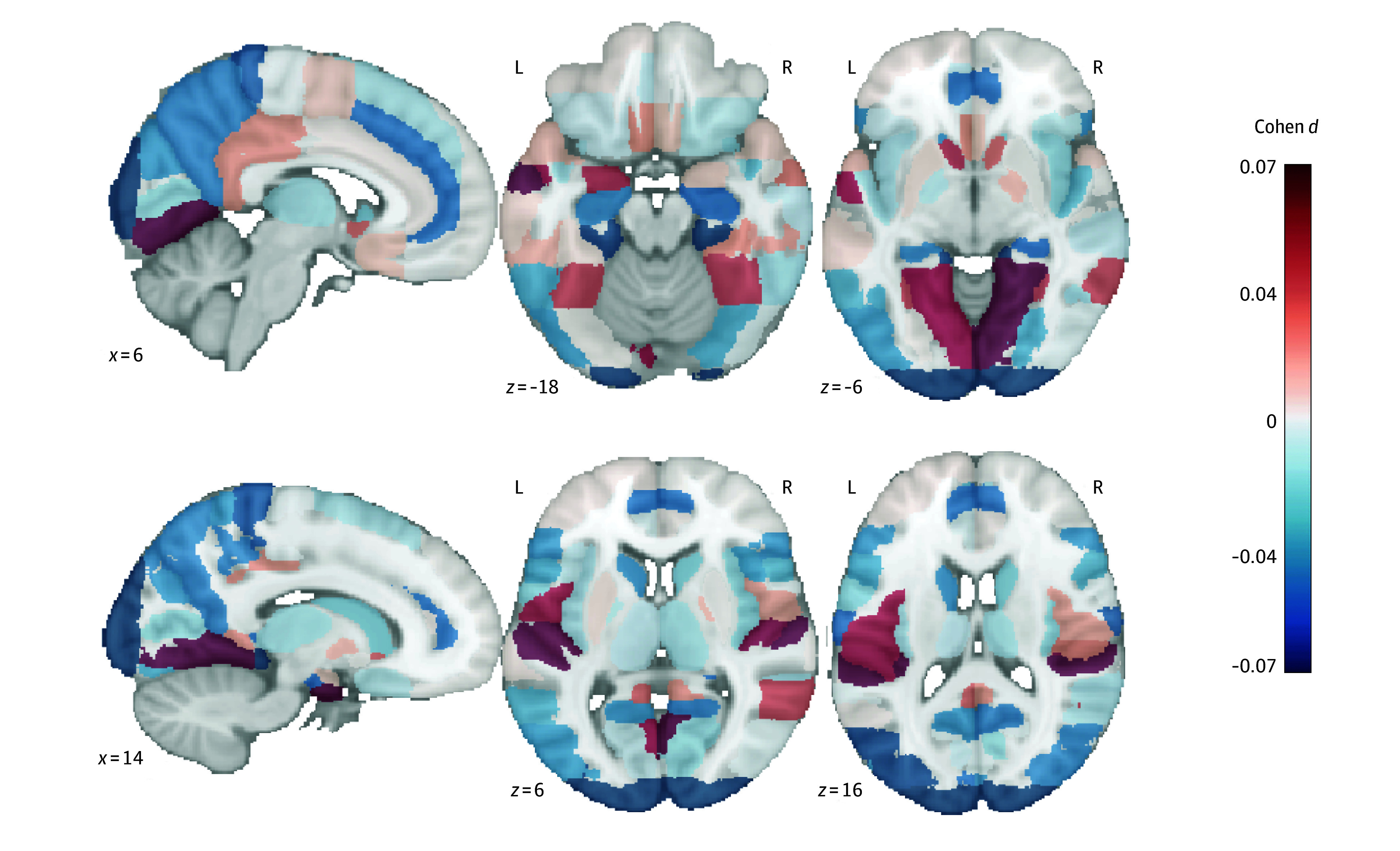
Brain Structure Associated With Travel Mode The neuroimaging analyses, after controlling for covariates and multiple comparisons, provided evidence for positive associations between cycling and mixed-cycling mode and 10 regional gray matter volumes. The negative values do not imply an increase in dementia risk but rather represent areas in which changes in brain volume were not associated with the active travel modes. L indicates left; R, right.

Among 44 801 participants with complete brain MRI data, neuroimaging analyses demonstrated significant associations between cycling and mixed-cycling mode and increased GMV in 10 brain regions (Cohen *d* = 0.004-0.108; *P* < .05) (Figure 2 and eTable 14 in Supplement 1) and 1 region after multiple comparisons. The negative values in Figure 2 do not imply an increase in dementia risk but rather represent areas in which changes in brain volume were not associated with the active travel modes. Additional associations of travel mode with representative brain structural measurements are shown in eTable 15 in Supplement 1. After adjustment for multiple potential confounders, travel mode was not associated with white matter hyperintensities. We observed that the cycling and mixed-cycling mode was significantly associated with a higher hippocampal volume (β, 0.05 [95% CI, 0.02-0.08). Conversely, walking and mixed-walking were significantly associated with a smaller volume of gray matter (β, –0.06 [95% CI, –0.09 to –0.03] for walking; β, –0.02 [95% CI. –0.04 to –0.01] for mixed-walking) and white matter (β, –0.02 [95% CI, –0.043 to –0.004 for mixed-walking). No significant structural changes were observed among nonactive travel subgroups (eTable 16 in Supplement 1).

Stratified analyses revealed no modification of the association between travel mode and YOD (*P* = .76 for interaction) or AD (*P* = .12 for interaction) by genetic susceptibility. However, genetic risk significantly modified the association with all-cause dementia (*P* = .02 for interaction) and LOD (*P* = .04 for interaction) (eTable 17 in Supplement 1). Specifically, for the cycling and mixed-cycling groups, the risk of all-cause dementia was lower among those without *APOE* ε4 (HR, 0.74 [95% CI, 0.63-0.87]) compared with those with *APOE* ε4 (HR, 0.88 [95% CI, 0.76-1.02]). Similarly, the risk of LOD was lower among those without *APOE* ε4 (HR, 0.75 [95% CI, 0.63-0.89]) compared with those with *APOE* ε4 (HR, 0.91 [95% CI, 0.78-1.05]).

Stratified analyses by sex and the Townsend Deprivation Index yielded results consistent with the main findings (eTable 18 in Supplement 1). The results remained robust for sensitivity analyses without adjusting for the IPAQ levels or without the younger than 60 years’ inclusion criterion for YOD (eTables 19 and 20 in Supplement 1). Analyses among employed participants also showed similar associations, after adjusting for the commuting mode, between travel mode and dementia risk (eTable 21 in Supplement 1). The results remained robust for the complete-case analysis and competing risk adjustments using the Fine and Gray model (eTables 22 and 23 in Supplement 1).

## Discussion

In this prospective cohort study, we identified an association between active travel modes and decreased risk of dementia. Cycling and mixed-cycling modes, in particular, were associated with a lower incidence of all-cause dementia, YOD, LOD, and AD. Additionally, these modes were positively associated with higher hippocampal volumes, suggesting potential neurobiologic mechanisms underlying these associations. Our findings suggest that promoting active travel strategies, particularly cycling, may be associated with lower dementia risk among middle-aged and older adults, which carries substantial public health benefits by encouraging accessible, sustainable practices for cognitive health preservation.

To our knowledge, limited evidence has explored the association between specific active travel modes and dementia risk. Although the health benefits of active travel for cardiometabolic conditions and mortality are well documented, primarily by increasing PA levels, evidence linking active travel specifically to dementia risk remains limited.^26^ A cross-sectional study involving 32 715 participants across 6 low-income and middle-income countries reported a higher risk of mild cognitive impairment with low active travel levels (odds ratio, 1.70 [95% CI, 1.32-2.19]) among individuals aged 65 years or older.^27^ In those younger than 65 years, no association was found (odds ratio, 1.07 [95% CI, 0.89-1.30]), yet our study observed significant results in relatively younger populations. The previous study categorized participants based on weekly active travel time without considering the differential factor of various travel modes,^27^ whereas our study enriched this evidence, highlighting the diverse effects of different travel modes, which not only vary in PA intensity but also in other health-related factors, such as cognitive demands and engagement. Studying individuals under the age of 60 years may have helped to further elucidate the specific risk factors, pathogenesis, and management approaches for YOD, and the results remained robust in sensitivity analyses for YOD without including the criterion for those younger than 60 years, providing a more comprehensive understanding of dementia across the entire age spectrum.^28^

Another interesting finding from our study was the lack of association between walking alone (ie, excluding mixed-walking) and a decreased risk of AD. Walking appeared to have a marginally increased risk tendency. However, a randomized clinical trial of a PA program demonstrated that exercise improves cognitive function in older adults, and the exercise group walked approximately 9000 additional steps per week compared with the control group, emphasizing the importance of walking distance and intensity.^29^ Furthermore, Schwenk et al found that complex task training (eg, walking while catching a ball or performing mental arithmetic) was associated with improved cognitive function more than simple walking tasks, suggesting that cognitive engagement during walking exercise is crucial for cognitive benefits.^30^ Our results suggest that mixed-walking models, which combine walking with other forms of travel that require higher cognitive engagement (eg, driving), may be more beneficial in reducing dementia risk than walking alone. Further categorization of nonactive travel modes revealed that car or motor vehicle use was associated with a significantly lower risk of all-cause dementia (HR, 0.78 [95% CI, 0.72-0.85]), potentially supporting this observation. Overall, the association of walking with brain health remains inconsistent and warrants further investigation. Our results further demonstrate that cycling and mixed-cycling might offer more benefits in preventing all-cause dementia, AD, YOD, and LOD than walking alone.

Our findings suggest that driving, relative to public transportation, might represent a more cognitively beneficial form of transportation. Research has shown that driving cessation is a major negative life event associated with declining overall health and cognitive function.^31,32^ Our study contributes to the growing evidence of a longitudinal association between driving cessation and increased dementia risk, consistent with prior research findings.^33,34^ Driving may support cognitive health by promoting an active life space and by engaging the brain. Basic research has shown that enriched environments, which include regular PA, foster brain plasticity through mechanisms such as synaptogenesis, neurogenesis, and attenuation of stress-related neural responses.^35,36,37^ Colcombe et al further demonstrated that PA is associated with increased blood perfusion of brain regions that modulate attention.^38^ Our study supports this notion, as travel modes with higher cognitive demands, such as driving and cycling, were positively associated with 10 regional GMVs. Although 1 study found that driving was associated with larger hippocampal volumes,^11^ our data did not support this association. However, cycling presented an association with increased hippocampal volumes. The finding that the mixed-cycling group was healthier at baseline and was likely to remain healthy in the years before and after cohort enrollment may suggest a potential mechanism for promoting cognitive health.^29^

Recognizing that some individuals with undiagnosed cognitive decline may have stopped driving or cycling before diagnosis, we restricted our analysis to employed participants to better understand the association of commuting mode or travel mode with incident dementia. Active travel modes showed decreased dementia risk tendency, and the consistency was observed even after further adjustment for commuting modes. Furthermore, we observed a significant interaction between travel mode and *APOE* ε4 status for all-cause dementia and LOD, prompting the gene-environment interaction in dementia occurrence. Cycling and mixed-cycling modes showed a significantly lower risk of all-cause dementia in the group without *APOE* ε4 (HR, 0.74 [95% CI, 0.63-0.87]) compared with the group with *APOE* ε4 (HR, 0.88 [95% CI, 0.76-1.02]), suggesting that those without *APOE* ε4 derived greater benefits from active travel modes.

### Strengths and Limitations

The strengths of this study include its comprehensive, novel insights concerning different travel modes, dementia risk, and brain structure changes. Additionally, the long-term follow-up, large sample size, and use of brain imaging data enhance the reliability and innovation of our findings.

However, several limitations warrant consideration. First, the low incidence of YOD restricted further categorizing cycling and mixed-cycling modes, resulting in reduced statistical power. Future research with longer follow-up periods and a larger number of participants with YOD is needed. Second, reliance on self-reported data for travel modes might have introduced measurement errors. Third, the heterogeneous clinical presentations of YOD compared with LOD posed challenges in accurate subtype classification, potentially leading to delayed diagnosis or misdiagnosis of participants with YOD. Fourth, we did not capture the long-term travel mode trajectories, and future studies with repeated measurements would provide valuable insights. Finally, our study cohort exhibited limited racial and ethnic diversity, with over 85% of individuals of European ancestry, necessitating caution when generalizing our findings to other racial and ethnic groups.

## Conclusions

The findings of this cohort study suggest that active travel modes, particularly cycling and mixed-cycling, are associated with a reduced incidence of dementia (YOD and LOD) and AD and greater hippocampal volume. These results may offer a promising approach to better brain health and lower dementia risk.

## Notes

Supplement 1. **eFigure 1.** Flow Chart of Participants Included in the Present UK Biobank Study **eFigure 2.** Associations of Travel Mode With Incident YOD, LOD, Dementia, and AD **eFigure 3.** Associations of Nonactive Travel Mode With Incident YOD, LOD, Dementia, and AD **eTable 1.** Disease Definitions for Dementia in UK Biobank Study **eTable 2.** Neuroimaging Markers Used in All Analyses in the Study **eTable 3.** Covariates Definitions in UK Biobank Study **eTable 4.** The Numbers (Percentages) of Participants With Missing Covariates **eTable 5.** Baseline Characteristics of Participants for YOD Analyses **eTable 6.** Baseline Characteristics of Participants for LOD Analyses **eTable 7.** Incidence Rate of Dementia in Different Travel Modes **eTable 8.** Association Between Travel Mode and Incident LOD **eTable 9.** Association Between Travel Mode and Incident YOD **eTable 10.** Association Between Travel Mode and Incident AD **eTable 11.** Association Between Nonactive Travel Mode and Incident LOD **eTable 12.** Association Between Nonactive Travel Mode and Incident AD **eTable 13.** Association Between Nonactive Travel Mode and Incident YOD, UK Biobank 2006 to 2010 **eTable 14.** Brain Regions Showing Significant Associations With Travel Mode (Cycling and Mixed-Cycling Mode) **eTable 15.** *z* Standardized Mean Differences and 95% CI in Brain Structure Measures According to Travel Mode (n = 44 988) **eTable 16.** *z* Standardized Mean Differences and 95% CI in Brain Structure Measures According to Nonactive Travel Mode (n = 44 988) **eTable 17.** Subgroup Analysis of the Association Between Travel Mode and the Risk of All-Cause Dementia, YOD, LOD, and AD by Genetic Risk **eTable 18.** Stratified Analysis for the Association Between Travel Mode and Incidence of All-Cause Dementia, YOD, LOD, and AD **eTable 19.** The Association of Travel Mode With Incident Dementia Without Adjusting for the IPAQ **eTable 20.** The Association of Travel Mode With Incident Dementia Without the Aged <60 Years’ Inclusion Criterion for YOD **eTable 21.** The Association of Commuting Mode or Travel Mode With Incident Dementia in Employed Participants **eTable 22.** Association Between Travel Mode and Incident All-Cause Dementia, AD, YOD, and LOD by Complete Cases **eTable 23.** Association Between Travel Mode and Incident All-Cause Dementia, AD, YOD, and LOD Using Fine and Gray Models for Competing Risk

Supplement 2.
Data Sharing Statement
