# Supplementary material for: Active Travel Mode and Incident Dementia and Brain Structure
Source: JAMA Netw Open. 2025 Jun 9;8(6):e2514316. doi: 10.1001/jamanetworkopen.2025.14316 (PMC12150188; doi:10.1001/jamanetworkopen.2025.14316)
Supplement: Supplement 1. — eFigure 1. Flow Chart of Participants Included in the Present UK Biobank Study eFigure 2. Associations of Travel Mode With Incident YOD, LOD, Dementia, and AD eFigure 3. Associations of Nonactive Travel Mode With Incident YOD, LOD, Dementia, and AD eTable 1. Disease Definitions for Dementia in UK Biobank Study eTable 2. Neuroimaging Markers Used in All Analyses in the Study eTable 3. Covariates Definitions in UK Biobank Study eTable 4. The Numbers (Percentages) of Participants With Missing Covariates eTable 5. Baseline Characteristics of Participants for YOD Analyses eTable 6. Baseline Characteristics of Participants for LOD Analyses eTable 7. Incidence Rate of Dementia in Different Travel Modes eTable 8. Association Between Travel Mode and Incident LOD eTable 9. Association Between Travel Mode and Incident YOD eTable 10. Association Between Travel Mode and Incident AD eTable 11. Association Between Nonactive Travel Mode and Incident LOD eTable 12. Association Between Nonactive Travel Mode and Incident AD eTable 13. Association Between Nonactive Travel Mode and Incident YOD, UK Biobank 2006 to 2010 eTable 14. Brain Regions Showing Significant Associations With Travel Mode (Cycling and Mixed-Cycling Mode) eTable 15. z Standardized Mean Differences and 95% CI in Brain Structure Measures According to Travel Mode (n = 44 988) eTable 16. z Standardized Mean Differences and 95% CI in Brain Structure Measures According to Nonactive Travel Mode (n = 44 988) eTable 17. Subgroup Analysis of the Association Between Travel Mode and the Risk of All-Cause Dementia, YOD, LOD, and AD by Genetic Risk eTable 18. Stratified Analysis for the Association Between Travel Mode and Incidence of All-Cause Dementia, YOD, LOD, and AD eTable 19. The Association of Travel Mode With Incident Dementia Without Adjusting for the IPAQ eTable 20. The Association of Travel Mode With Incident Dementia Without the Aged <60 Years’ Inclusion Criterion for YOD eTable 21. The Association of Commuting Mode or [file jamanetwopen-e2514316-s001.pdf]

Supplemental Online Content

Hou C, Zhang Y, Zhao F, et al. Active travel mode and incident dementia and brain structure. *JAMA Netw Open*. 2025;8(6):e2514316. doi:10.1001/jamanetworkopen.2025.14316

eFigure 1. Flow Chart of Participants Included in the Present UK Biobank Study
eFigure 2. Associations of Travel Mode With Incident YOD, LOD, Dementia, and AD
eFigure 3. Associations of Nonactive Travel Mode With Incident YOD, LOD, Dementia, and AD
eTable 1. Disease Definitions for Dementia in UK Biobank Study
eTable 2. Neuroimaging Markers Used in All Analyses in the Study
eTable 3. Covariates Definitions in UK Biobank Study
eTable 4. The Numbers (Percentages) of Participants With Missing Covariates
eTable 5. Baseline Characteristics of Participants for YOD Analyses
eTable 6. Baseline Characteristics of Participants for LOD Analyses
eTable 7. Incidence Rate of Dementia in Different Travel Modes
eTable 8. Association Between Travel Mode and Incident LOD
eTable 9. Association Between Travel Mode and Incident YOD
eTable 10. Association Between Travel Mode and Incident AD
eTable 11. Association Between Nonactive Travel Mode and Incident LOD
eTable 12. Association Between Nonactive Travel Mode and Incident AD
eTable 13. Association Between Nonactive Travel Mode and Incident YOD, UK Biobank 2006 to 2010
eTable 14. Brain Regions Showing Significant Associations With Travel Mode (Cycling and Mixed-Cycling Mode)
eTable 15. *z* Standardized Mean Differences and 95% CI in Brain Structure Measures According to Travel Mode (n = 44 988)
eTable 16. *z* Standardized Mean Differences and 95% CI in Brain Structure Measures According to Nonactive Travel Mode (n = 44 988)
eTable 17. Subgroup Analysis of the Association Between Travel Mode and the Risk of All-Cause Dementia, YOD, LOD, and AD by Genetic Risk
eTable 18. Stratified Analysis for the Association Between Travel Mode and Incidence of All-Cause Dementia, YOD, LOD, and AD
eTable 19. The Association of Travel Mode With Incident Dementia Without Adjusting for the IPAQ
eTable 20. The Association of Travel Mode With Incident Dementia Without the Aged <60 Years' Inclusion Criterion for YOD
eTable 21. The Association of Commuting Mode or Travel Mode With Incident Dementia in Employed Participants
eTable 22. Association Between Travel Mode and Incident All-Cause Dementia, AD, YOD, and LOD by Complete Cases
eTable 23. Association Between Travel Mode and Incident All-Cause Dementia, AD, YOD, and LOD Using Fine and Gray Models for Competing Risk

This supplemental material has been provided by the authors to give readers additional information about their work.

© 2025 Hou C et al. *JAMA Network Open*.

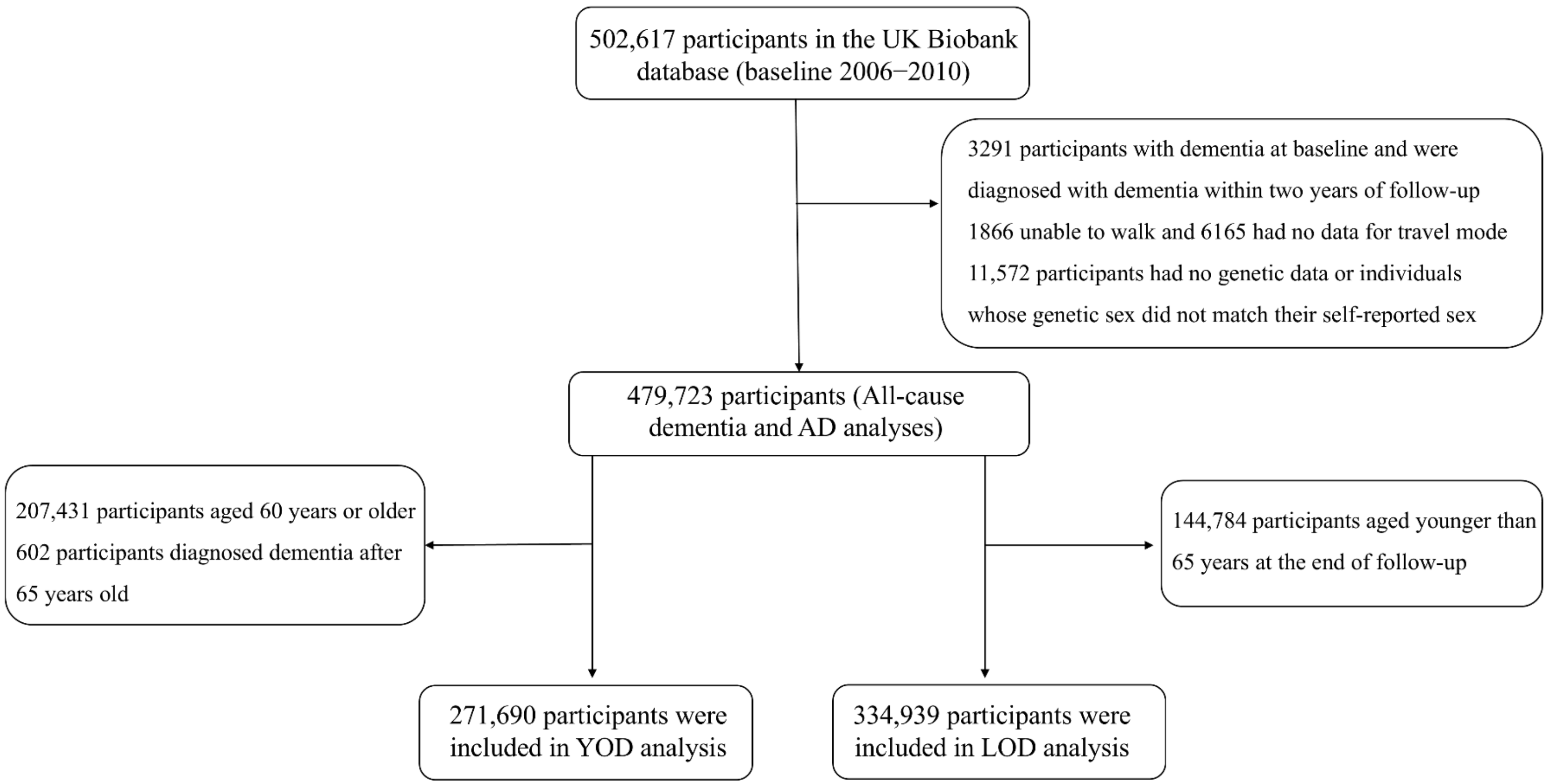

eFigure 1. Flow Chart of Participants Included in the Present UK Biobank Study

© 2025 Hou C et al. *JAMA Network Open*.

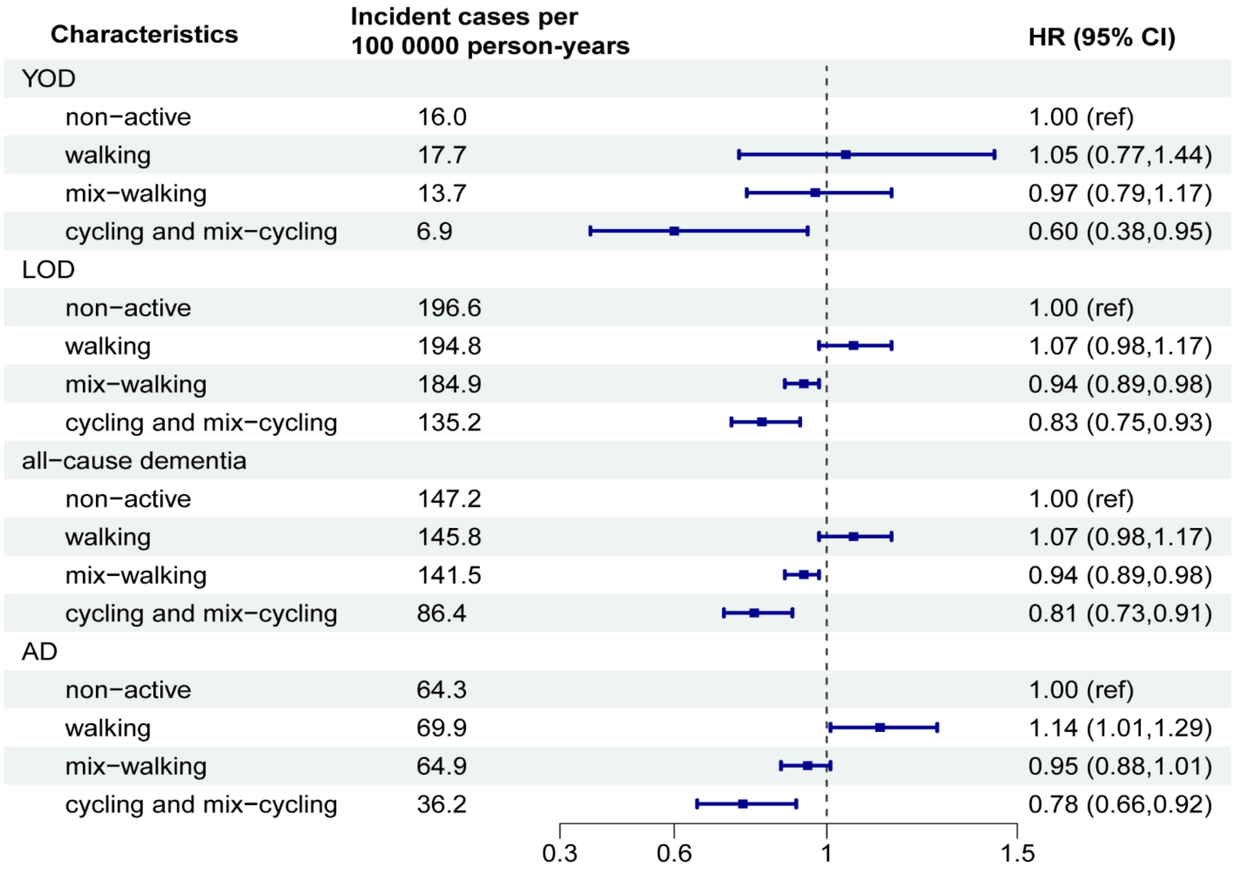

eFigure 2. Associations of Travel Mode With Incident YOD, LOD, Dementia, and AD

© 2025 Hou C et al. *JAMA Network Open*.

Characteristics	Incident cases per 100 0000 person-years	HR (95% CI)
YOD		
only public transporter	38.0	1.00 (ref)
only car/motor	13.1	0.73 (0.53,1.01)
public transporter+car/motor	19.5	0.87 (0.50,1.49)
LOD		
only public transporter	333.3	1.00 (ref)
only car/motor	166.3	0.79 (0.73,0.86)
public transporter+car/motor	260.7	0.82 (0.73,0.91)
all−cause dementia		
only public transporter	267.0	1.00 (ref)
only car/motor	121.9	0.78 (0.72,0.85)
public transporter+car/motor	217.7	0.81 (0.73,0.91)
AD		
only public transporter	115.8	1.00 (ref)
only car/motor	53.4	0.84 (0.75,0.95)
public transporter+car/motor	95.7	0.83 (0.71,0.99)

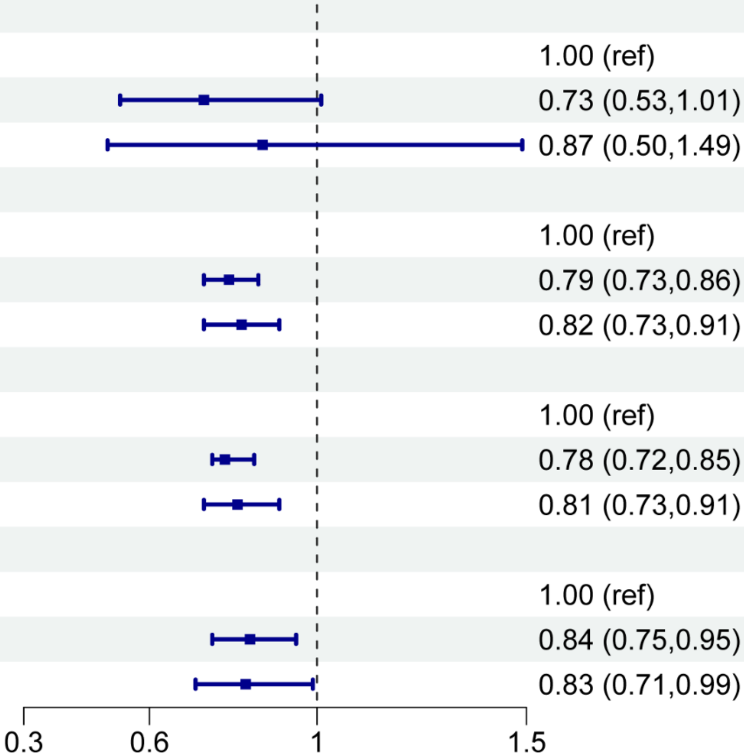

eFigure 3. Associations of Nonactive Travel Mode With Incident YOD, LOD, Dementia, and AD

© 2025 Hou C et al. *JAMA Network Open*.

eTable 1. Disease Definitions for Dementia in UK Biobank Study

	**ICD-9**	**ICD-10**	**Self-reported fields**
**All-cause dementia**	2902, 2903, 2904, 2912, 2941, 3310, 3311, 3312, 3315	A81.0, F00-F03, F05.1, F10.6, G30, G31, I67.3	20002
**AD**	3310	F00, G30	

Abbreviations: ICD, International Classification of Diseases; AD, Alzheimer's disease.

© 2025 Hou C et al. *JAMA Network Open*.

eTable 2. Neuroimaging Markers Used in All Analyses in the Study

**Neuroimaging markers**		
**Variable**	**Field ID**	**UK Biobank showcase link**
**Head scaling factor**	25000	https://biobank.ndph.ox.ac.uk/showcase/field.cgi?id=25000
**Cortical volume**	25782-25877	https://biobank.ndph.ox.ac.uk/showcase/label.cgi?id=1101
**Subcortical volume**	25011-25024	https://biobank.ndph.ox.ac.uk/showcase/label.cgi?id=1102

© 2025 Hou C et al. *JAMA Network Open*.

eTable 3. Covariates Definitions in UK Biobank Study

Variable (UKB ID)	Collection	Description	Link
Age (21003)	Registry	Participant age at assessment centre visit, in years, obtained from NHS Primary Care Trust registries. Confirmed with participants at assessment centre.	https://biobank.ndph.ox.ac.uk/showcase/field.cgi?id=21003
Sex (31)	Registry	From NHS Primary Care Trust registries. Confirmed with participants at assessment centre.	https://biobank.ndph.ox.ac.uk/showcase/field.cgi?id=31
Ethnic background (21000)	Assessment centre visit: Questionnaire	Ethnic group (white, mixed, Asian/Asian British, Black/Black British, Chinese, other, PNTA) and ethnic background sub-categories.	https://biobank.ndph.ox.ac.uk/showcase/field.cgi?id=21000
Townsend deprivation index (189)	Registry	Scores represent deprivation by local area, quantified by average home ownership, car ownership, household overcrowding, and employment rate, and were derived using national census data at time of recruitment. Higher scores indicate more deprived.	https://biobank.ndph.ox.ac.uk/showcase/field.cgi?id=189
Smoking status (20116)	Assessment centre visit: Questionnaire	Smoking status / history: never, previous, current, PNTA	https://biobank.ndph.ox.ac.uk/showcase/field.cgi?id=20116
Alcohol intake frequency (1558)	Assessment centre visit: Questionnaire	Daily, 3-4 times per week, 1-2 times per week, 1-3 times per month, special occasions only, never, PNTA	https://biobank.ndph.ox.ac.uk/showcase/field.cgi?id=1558

© 2025 Hou C et al. *JAMA Network Open*.

Body mass index (21001)	Assessment centre visit: Physical measurements	Weight (kg) / height (m)^2	https://biobank.ndph.ox.ac.uk/showcase/field.cgi?id=21001
Physical activity (22032)	Assessment centre visit: Questionnaire	Low, Moderate, High	https://biobank.ndph.ox.ac.uk/showcase/field.cgi?id=22032
Assessment centers (54)	Registry	UK Biobank assessment center	https://biobank.ndph.ox.ac.uk/showcase/field.cgi?id=54
Current employment status (6142)	Assessment centre visit: Questionnaire	Paid employment, unemployed, retired, home/family caretaker, unable to work, volunteer, student, other	https://biobank.ndph.ox.ac.uk/showcase/field.cgi?id=6142
Reaction time (20023)	Assessment centre visit: Cognitive function	Mean time to correctly identify matches, and longer reaction time means worse cognitive function.	https://biobank.ndph.ox.ac.uk/showcase/field.cgi?id=20023
Long-standing illness, disability or infirmity (2188)	Assessment centre visit: Questionnaire	Yes, NO, Don't know, PNTA	https://biobank.ndph.ox.ac.uk/showcase/field.cgi?id=2188
Baseline of diabetes	ICD-10: E10, E11, E12, E13, E14<br>ICD-9: 250, 6480<br>Self-reported field IDs: 2443<br>Illness code: 1220, 1222,1223		
Baseline of cardiovascular disease	ICD-10: I20-25, I50, I500, I501, I509, I60, I61, I63, I64<br>ICD-9: 410, 411, 412, 413, 414, 428, 4280, 4281,4289, 430, 431, 434, 4340, 4341, 4349, 436<br>Self-reported field IDs: 6150, 3894, 3627, 20002, 20004, 4056		
Baseline of depression	ICD-10: F32, F33, F34, F38, F39<br>ICD-9: 2962, 2963, 2969, 3004, 3119<br>Illness code: 1286, 1531		

© 2025 Hou C et al. *JAMA Network Open*.

Baseline of hypertension	ICD-10: I10, I11, I12, I13, I15, O10, O11<br>ICD-9: 401, 402, 403, 404, 405<br>Self-reported field IDs: 6150<br>Illness code: 1065, 1072
Baseline of dyslipidemia	ICD-10: E78<br>ICD-9: 2720, 2721, 2722, 2723, 2724, 2725, 2726, 2727, 2728, 2729<br>Self-reported field IDs: 1473

Abbreviations: PNTA, prefer not to answer

© 2025 Hou C et al. *JAMA Network Open*.

eTable 4. The Numbers (Percentages) of Participants With Missing Covariates

Covariates	n	%
Ethnicity	1576	0.33%
Education	4993	1.04%
Townsend deprivation index	589	0.12%
Smoking status	1796	0.37%
Alcohol consumption	463	0.10%
IPAQ activity group	90919	18.95%
BMI	1533	0.32%
Reaction time	4391	0.92%

© 2025 Hou C et al. *JAMA Network Open*.

eTable 5. Baseline Characteristics of Participants for YOD Analyses

Baseline characteristics	Travel mode, No. (%)			
	Non-active	Walking	mixed-walking	cycling and mixed-cycling
No. of participants	135772 (50.0)	19927 (7.3)	93530 (34.4)	22461 (8.3)
Age, mean (SD), years	50.8 (5.6)	50.9 (5.6)	50.7 (5.6)	49.7 (5.6)
Sex				
Male	61049 (45.0)	8290 (41.6)	37668 (40.3)	13693 (61.0)
Female	74723 (55.0)	11637 (58.4)	55862 (59.7)	8768 (39.0)
Ethnicity				
White	124179 (91.5)	18167 (91.2)	87668 (93.7)	21564 (96.0)
Non-White	11191 (8.2)	1659 (8.3)	5586 (6.0)	811 (3.6)
Unknown	402 (0.3)	101 (0.5)	276 (0.3)	86 (0.4)
Education				
College or University	45592 (33.6)	7007 (35.2)	37488 (40.1)	11877 (52.9)
Vocational	15486 (11.4)	2245 (11.3)	7643 (8.2)	1621 (7.2)
Upper secondary	16463 (12.1)	2194 (11.0)	13603 (14.5)	2756 (12.3)
Lower secondary	41555 (30.6)	5595 (28.1)	27664 (29.6)	5014 (22.3)
Others	15369 (11.3)	2631 (13.2)	6560 (7.0)	1082 (4.8)
Unknown	1307 (1.0)	255 (1.3)	572 (0.6)	111 (0.5)
Townsend deprivation index, median (IQR)	-2.2 (-3.7, 0.6)	-0.7 (-2.9, 2.2)	-1.9 (-3.5, 0.8)	-1.8 (-3.5, 0.8)
Smoking status				
Never	77925 (57.4)	11066 (55.5)	56510 (60.4)	13040 (58.1)
Former	39545 (29.1)	5803 (29.1)	26801 (28.7)	7156 (31.9)
Current	17867 (13.2)	2983 (15.0)	9994 (10.7)	2222 (9.9)
Alcohol consumption				
Daily or almost daily	23702 (17.5)	3523 (17.7)	16972 (18.1)	4915 (21.9)
3 or 4 Times a week	31187 (23.0)	4287 (21.5)	23187 (24.8)	6521 (29.0)
Once or twice a week	37337 (27.5)	5224 (26.2)	25695 (27.5)	6021 (26.8)
1-3 Times a month	16612 (12.2)	2355 (11.8)	11587 (12.4)	2288 (10.2)
Never or special occasions only	26786 (19.7)	4498 (22.6)	16013 (17.1)	2697 (12.0)
IPAQ activity group				
Low	29929 (22.0)	1717 (8.6)	11908 (12.7)	1181 (5.3)
Moderate	42044 (31.0)	6865 (34.5)	34891 (37.3)	6015 (26.8)
High	37885 (27.9)	8070 (40.5)	31259 (33.4)	12680 (56.5)
Unknown	25914 (19.1)	3275 (16.4)	15472 (16.5)	2585 (11.5)
BMI, mean (SD)	27.8 (5.2)	26.5 (4.6)	27.0 (4.8)	25.8 (3.9)
Occupation				

© 2025 Hou C et al. *JAMA Network Open*.

Employed	112261 (82.7)	15076 (75.7)	74035 (79.2)	19056 (84.8)
Unemployed	23511 (17.3)	4851 (24.3)	19495 (20.8)	3405 (15.2)
Diabetes	7049 (5.2)	883 (4.4)	3848 (4.1)	528 (2.4)
Dyslipidemia	68318 (50.3)	8865 (44.5)	43115 (46.1)	8850 (39.4)
Hypertension	62250 (45.8)	9053 (45.4)	41465 (44.3)	8385 (37.3)
CVD	5644 (4.2)	797 (4.0)	3244 (3.5)	515 (2.3)
Depression	17011 (12.5)	2496 (12.5)	11740 (12.6)	2270 (10.1)
Long-standing illness, disability or infirmity	39086 (28.8)	5353 (26.9)	25193 (26.9)	4679 (20.8)
Reaction time, mean (SD), ms	537.0 (110.3)	542.7 (114.3)	534.3 (104.4)	519.9 (97.5)
APOE ε4 carriers	38544 (28.4)	5634 (28.3)	26985 (28.9)	6519 (29.0)

© 2025 Hou C et al. *JAMA Network Open*.

eTable 6. Baseline Characteristics of Participants for LOD Analyses

Baseline characteristics	Travel mode, No. (%)			
	Non-active	Walking	mixed-walking	cycling and mix-cycling
No. of participants	163846 (48.9)	22539 (6.7)	128207 (38.3)	20347 (6.1)
Age, mean (SD), years	60.8 (5.0)	60.4 (4.9)	61.2 (4.9)	60.1 (5.0)
Sex				
Male	75511 (46.1)	10154 (45.1)	54669 (42.6)	13084 (64.3)
Female	88335 (53.9)	12385 (54.9)	73538 (57.4)	7263 (35.7)
Ethnicity				
White	156396 (95.5)	21449 (95.2)	123799 (96.6)	19936 (98.0)
Non-White	7007 (4.3)	990 (4.4)	3953 (3.1)	320 (1.6)
Unknown	443 (0.3)	100 (0.4)	455 (0.4)	91 (0.4)
Education				
College or University	41976 (25.6)	6508 (28.9)	42366 (33.0)	9295 (45.7)
Vocational	23893 (14.6)	3148 (14.0)	15248 (11.9)	2347 (11.5)
Upper secondary	14692 (9.0)	2061 (9.1)	15055 (11.7)	2156 (10.6)
Lower secondary	39631 (24.2)	4951 (22.0)	31858 (24.8)	3787 (18.6)
Others	41553 (25.4)	5521 (24.5)	22533 (17.6)	2606 (12.8)
Unknown	2101 (1.3)	350 (1.6)	1147 (0.9)	156 (0.8)
Townsend deprivation index, median (IQR)	-2.4 (-3.8, 0.1)	-1.5 (-3.2, 1.4)	-2.3 (-3.7, 0.0)	-2.3 (-3.7, 0.1)
Smoking status				
Never	82359 (50.3)	11712 (52.0)	70052 (54.6)	10860 (53.4)
Former	64098 (39.1)	8441 (37.5)	48027 (37.5)	8106 (39.8)
Current	16601 (10.1)	2265 (10.0)	9685 (7.6)	1320 (6.5)
Alcohol consumption				
Daily or almost daily	35109 (21.4)	4842 (21.5)	29195 (22.8)	5754 (28.3)
3 or 4 Times a week	36273 (22.1)	4919 (21.8)	30761 (24.0)	5633 (27.7)
Once or twice a week	41025 (25.0)	5592 (24.8)	31303 (24.4)	4709 (23.1)
1-3 Times a month	16933 (10.3)	2274 (10.1)	13427 (10.5)	1711 (8.4)
Never or special occasions only	34363 (21.0)	4885 (21.7)	23435 (18.3)	2532 (12.4)
IPAQ activity group				
Low	32476 (19.8)	1630 (7.2)	13964 (10.9)	948 (4.7)
Moderate	50708 (30.9)	7802 (34.6)	47331 (36.9)	5571 (27.4)
High	43795 (26.7)	9245 (41.0)	42838 (33.4)	11203 (55.1)
Unknown	36867 (22.5)	3862 (17.1)	24074 (18.8)	2625 (12.9)
BMI, mean (SD)	28.1 (4.9)	26.5 (4.2)	27.2 (4.4)	26.0 (3.7)
Occupation				

© 2025 Hou C et al. *JAMA Network Open*.

Employed	79625 (48.6)	10502 (46.6)	51354 (40.1)	10652 (52.4)
Unemployed	84221 (51.4)	12037 (53.4)	76853 (59.9)	9695 (47.6)
Diabetes	13470 (8.2)	1293 (5.7)	8154 (6.4)	768 (3.8)
Dyslipidemia	98164 (59.9)	11649 (51.7)	71664 (55.9)	9712 (47.7)
Hypertension	106224 (64.8)	14161 (62.8)	82013 (64.0)	11354 (55.8)
CVD	16453 (10.0)	1742 (7.7)	10624 (8.3)	1216 (6.0)
Depression	17722 (10.8)	2269 (10.1)	13402 (10.5)	1849 (9.1)
Long-standing illness, disability or infirmity	60581 (37.0)	6902 (30.6)	41857 (32.6)	5411 (26.6)
Reaction time, mean (SD), ms	575.8 (120.2)	578.4 (122.7)	575.8 (116.0)	560.0 (110.3)
APOE ε4 carriers	45978 (28.1)	6375 (28.3)	36424 (28.4)	5840 (28.7)

eTable 7. Incidence Rate of Dementia in Different Travel Modes

Cases/N (%)	All-cause dementia	AD	YOD	LOD
Total	8845/479723 (1.84%)	3956/47572 3 (0.83%)	528/271690 (0.19%)	8276/334939 (2.47%)
Non-active	4532/235508 (1.92%)	1981/23550 8 (0.84%)	289/135772 (0.21%)	4219/163846 (2.57%)
Walking	628/32836 (1.91%)	301/32836 (0.92%)	47/19927 (0.24%)	578/22539 (2.56%)
Mixed-walking	3296/177635 (1.86%)	1511/177635 (0.85%)	171/93530 (0.18%)	3111/128027 (2.43%)
Cycling and mixed-cycling	389/33744 (1.15%)	163/33744 (0.48%)	21/22461 (0.09%)	368/20347 (1.81%)

© 2025 Hou C et al. *JAMA Network Open*.

eTable 8. Association Between Travel Mode and Incident LOD

Characteristic	Non-active	Walking	Mixed-walking	Cycling and mixed-cycling
Cases/person-years	4219/2146148	578/296767	3111/1682855	368/272133
Incident cases per 100,000 person-years	196.6	194.8	184.9	135.2
Model 1, [a]HR (95% CI)	1.00 (ref.)	1.01 (0.93, 1.11)	**0.89 (0.85, 0.93)**	**0.74 (0.67, 0.83)**
Model 2, [b]HR (95% CI)	1.00 (ref.)	1.04 (0.95, 1.14)	**0.92 (0.87, 0.96)**	**0.78 (0.70, 0.88)**
Model 3, [c]HR (95% CI)	1.00 (ref.)	1.07 (0.98, 1.17)	**0.94 (0.89, 0.98)**	**0.83 (0.75, 0.93)**

[a]Model 1, adjusted for age, sex, race, education, Townsend deprivation index, employment status, and assessment centers.
[b]Model 2, included model 1 plus alcohol consumption, smoking status, BMI, and IPAQ activity group.
[c]Model 3, included model 2 plus diabetes, dyslipidemia, hypertension, CVD, depression, long-standing illness, disability or infirmity, cognitive function (i.e., reaction time), and genetic variables (APOE ε4 carrier status).

© 2025 Hou C et al. *JAMA Network Open.*

eTable 9. Association Between Travel Mode and Incident YOD

Characteristic	Non-active	Walking	Mixed-walking	Cycling and mixed-cycling
Cases/person-years	289/1808760	47/265330	171/1247212	21/302703
Incident cases per 100,000 person-years	16.0	17.7	13.7	6.9
Model 1, [a]HR (95% CI)	1.00 (ref.)	0.92 (0.67, 1.25)	0.87 (0.72, 1.06)	**0.48 (0.30, 0.74)**
Model 2, [b]HR (95% CI)	1.00 (ref.)	0.98 (0.72, 1.35)	0.93 (0.76, 1.13)	**0.54 (0.34, 0.85)**
Model 3, [c]HR (95% CI)	1.00 (ref.)	1.05 (0.77, 1.44)	0.97 (0.79, 1.17)	**0.60 (0.38, 0.95)**

[a]Model 1, adjusted for age, sex, race, education, Townsend deprivation index, employment status, and assessment centers.
[b]Model 2, included model 1 plus alcohol consumption, smoking status, BMI, and IPAQ activity group.
[c]Model 3, included model 2 plus diabetes, dyslipidemia, hypertension, CVD, depression, long-standing illness, disability or infirmity, cognitive function (i.e., reaction time), and genetic variables (APOE ε4 carrier status).

© 2025 Hou C et al. *JAMA Network Open.*

eTable 10. Association Between Travel Mode and Incident AD

Characteristic	Non-active	Walking	Mixed-walking	Cycling and mixed-cycling
Cases/person-years	1981/3079442	301/430638	1511/2328850	163/450156
Incident cases per 100,000 person-years	64.3	69.9	64.9	36.2
Model 1, [a]HR (95% CI)	1.00 (ref.)	**1.14 (1.01, 1.29)**	**0.93 (0.87, 1.00)**	**0.75 (0.64, 0.88)**
Model 2, [b]HR (95% CI)	1.00 (ref.)	1.12 (0.99, 1.27)	0.94 (0.87, 1.00)	**0.75 (0.63, 0.88)**
Model 3, [c]HR (95% CI)	1.00 (ref.)	**1.14 (1.01, 1.29)**	0.95 (0.88, 1.01)	**0.78 (0.66, 0.92)**

[a]Model 1, adjusted for age, sex, race, education, Townsend deprivation index, employment status, and assessment centers.
[b]Model 2, included model 1 plus alcohol consumption, smoking status, BMI, and IPAQ activity group.
[c]Model 3, included model 2 plus diabetes, dyslipidemia, hypertension, CVD, depression, long-standing illness, disability or infirmity, cognitive function (i.e., reaction time), and genetic variables (APOE ε4 carrier status).

© 2025 Hou C et al. *JAMA Network Open*.

eTable 11. Association Between Nonactive Travel Mode and Incident LOD

Characteristic	Only public transporter	Only car/motor	Public transporter+ car/motor
Cases/person-years	944/283215	2788/1676137	487/186796
Incident cases per 100,000 person-years	333.3	166.3	260.7
Model 1, [a]HR (95% CI)	1.00 (ref.)	**0.76 (0.70, 0.82)**	**0.79 (0.71, 0.88)**
Model 2, [b]HR (95% CI)	1.00 (ref.)	**0.77 (0.71, 0.83)**	**0.79 (0.71, 0.89)**
Model 3, [c]HR (95% CI)	1.00 (ref.)	**0.79 (0.73, 0.86)**	**0.82 (0.73, 0.91)**

[a]Model 1, adjusted for age, sex, race, education, Townsend deprivation index, employment status, and assessment centers.
[b]Model 2, included model 1 plus alcohol consumption, smoking status, BMI, and IPAQ activity group.
[c]Model 3, included model 2 plus diabetes, dyslipidemia, hypertension, CVD, depression, long-standing illness, disability or infirmity, cognitive function (i.e., reaction time), and genetic variables (APOE $\varepsilon 4$ carrier status).

© 2025 Hou C et al. *JAMA Network Open*.

eTable 12. Association Between Nonactive Travel Mode and Incident AD

Characteristic	Only public transporter	Only car/motor	Public transporter+ car/motor
Cases/person-years	443/382701	1316/2464786	222/231955
Incident cases per 100,000 person-years	115.8	53.4	95.7
Model 1, [a]HR (95% CI)	1.00 (ref.)	**0.81 (0.72, 0.91)**	**0.79 (0.67, 0.94)**
Model 2, [b]HR (95% CI)	1.00 (ref.)	**0.83 (0.73, 0.93)**	**0.81 (0.69, 0.96)**
Model 3, [c]HR (95% CI)	1.00 (ref.)	**0.84 (0.75, 0.95)**	**0.83 (0.71, 0.99)**

[a]Model 1, adjusted for age, sex, race, education, Townsend deprivation index, employment status, and assessment centers.
[b]Model 2, included model 1 plus alcohol consumption, smoking status, BMI, and IPAQ activity group.
[c]Model 3, included model 2 plus diabetes, dyslipidemia, hypertension, CVD, depression, long-standing illness, disability or infirmity, cognitive function (i.e., reaction time), and genetic variables (APOE ε4 carrier status).

© 2025 Hou C et al. *JAMA Network Open*.

eTable 13. Association Between Nonactive Travel Mode and Incident YOD, UK Biobank 2006 to 2010

Characteristic	Only public transporter	Only car/motor	Public transporter+ car/motor
Cases/person-years	70/184437	202/1537009	17/87313
Incident cases per 100,000 person-years	38.0	13.1	19.5
Model 1, [a]HR (95% CI)	1.00 (ref.)	**0.59 (0.43, 0.81)**	0.80 (0.47, 1.37)
Model 2, [b]HR (95% CI)	1.00 (ref.)	**0.61 (0.44, 0.83)**	0.80 (0.46, 1.37)
Model 3, [c]HR (95% CI)	1.00 (ref.)	0.73 (0.53, 1.01)	0.87 (0.50, 1.49)

[a]Model 1, adjusted for age, sex, race, education, Townsend deprivation index, employment status, and assessment centers.
[b]Model 2, included model 1 plus alcohol consumption, smoking status, BMI, and IPAQ activity group.
[c]Model 3, included model 2 plus diabetes, dyslipidemia, hypertension, CVD, depression, long-standing illness, disability or infirmity, cognitive function (i.e., reaction time), and genetic variables (APOE ε4 carrier status).

© 2025 Hou C et al. *JAMA Network Open.*

eTable 13. Association Between Nonactive Travel Mode and Incident AD

Characteristic	Only public transporter	Only car/motor	Public transporter+ car/motor
Cases/person-years	443/382701	1316/2464786	222/231955
Incident cases per 100,000 person-years	115.8	53.4	95.7
Model 1, [a]HR (95% CI)	1.00 (ref.)	**0.81 (0.72, 0.91)**	**0.79 (0.67, 0.94)**
Model 2, [b]HR (95% CI)	1.00 (ref.)	**0.83 (0.73, 0.93)**	**0.81 (0.69, 0.96)**
Model 3, [c]HR (95% CI)	1.00 (ref.)	**0.84 (0.75, 0.95)**	**0.83 (0.71, 0.99)**

[a]Model 1, adjusted for age, sex, race, education, Townsend deprivation index, employment status, and assessment centers.
[b]Model 2, included model 1 plus alcohol consumption, smoking status, BMI, and IPAQ activity group.
[c]Model 3, included model 2 plus diabetes, dyslipidemia, hypertension, CVD, depression, long-standing illness, disability or infirmity, cognitive function (i.e., reaction time), and genetic variables (APOE ε4 carrier status).

© 2025 Hou C et al. *JAMA Network Open*.

eTable 14. Brain Regions Showing Significant Associations With Travel Mode (Cycling and Mixed-Cycling Mode)

**Brain region**	**Coeff**	**se**	***P*-value**	***P*-FDR**	**T value**	**Cohen's d**
Right_Occipital_Pole	-0.062	0.0172	0.0003	**0.0156**	-3.61	-0.076
Left_Occipital_Pole	-0.047	0.0169	0.0052	0.1173	-2.79	-0.089
Right_Planum_Polare	-0.035	0.0159	0.0286	0.2076	-2.19	-0.035
Right_Temporal_Fusiform_Cortex_anterior_division	0.036	0.0173	0.0391	0.2352	2.06	0.093
Right_Middle_Temporal_Gyrus_temporooccipital_part	0.038	0.0177	0.0333	0.2170	2.13	0.042
Left_Superior_Temporal_Gyrus_anterior_division	0.038	0.0174	0.0292	0.2076	2.18	0.055
Left_Planum_Temporale	0.039	0.0173	0.0258	0.2076	2.23	0.103
Right_Juxtapositional_Lobule_Cortex	0.039	0.0173	0.0256	0.2076	2.23	0.012
Left_Juxtapositional_Lobule_Cortex	0.042	0.0174	0.0154	0.1721	2.42	0.004
Left_Central_Opercular_Cortex	0.042	0.0164	0.0095	0.1277	2.59	0.052
Left_Amygdala	0.046	0.0179	0.0098	0.1277	2.58	0.049
Right_Lingual_Gyrus	0.047	0.0173	0.0060	0.1173	2.75	0.074
Left_Temporal_Fusiform_Cortex_anterior_division	0.061	0.0171	0.0004	**0.0156**	3.57	0.108

© 2025 Hou C et al. *JAMA Network Open*.

eTable 15. *z* Standardized Mean Differences and 95% CI in Brain Structure Measures According to Travel Mode (n = 44 988)

	Non-active mode	Walking	Mixed-walking	Cycling and mixed-cycling
White matter hyperintensity (mm^3)	0 (ref.)	0.022 (-0.013, 0.056)	-0.006 (-0.024, 0.011)	-0.001 (-0.031, 0.029)
Gray matter volume (mm^3)	0 (ref.)	**-0.058 (-0.087, -0.028)**	**-0.021 (-0.036, -0.006)**	0.009 (-0.017, 0.035)
White matter volume (mm^3)	0 (ref.)	-0.022 (-0.060, 0.016)	**-0.024 (-0.043, -0.004)**	0.004 (-0.030, 0.037)
Peripheral cortical gray matter volume (mm^3)	0 (ref.)	**-0.064 (-0.095, -0.034)**	**-0.025 (-0.041, -0.010)**	0.003 (-0.024, 0.030)
Ventricular cerebrospinal fluid volume (mm^3)	0 (ref.)	**0.053 (0.018, 0.088)**	**0.026 (0.008, 0.043)**	**0.052 (0.022, 0.083)**
Hippocampal volume (mm^3)	0 (ref.)	-0.022 (-0.059, 0.016)	-0.007 (-0.026, 0.012)	**0.051 (0.018, 0.084)**
Subcortical volumes (mm^3)	0 (ref.)	-0.002 (-0.035, 0.032)	-0.013 (-0.030, 0.004)	**0.067 (0.038, 0.096)**
Total volume (mm^3)	0 (ref.)	**-0.050 (-0.083, -0.018)**	**-0.027 (-0.044, -0.010)**	0.008 (-0.020, 0.037)

Note: Z-standardized mean differences were calculated by linear regression models adjusted for age, sex, race, education, Townsend deprivation index, assessment centers, alcohol consumption, smoking status, BMI, IPAQ activity group, employment status, diabetes, dyslipidemia, hypertension, CVD, depression, long-standing illness, disability or infirmity, cognitive function (i.e., reaction time), and genetic variables (APOE ε4 carrier status). The volume of white matter hyperintensity was log-transformed before z-standardization because of skew distribution.

© 2025 Hou C et al. *JAMA Network Open*.

eTable 16. *z* Standardized Mean Differences and 95% CI in Brain Structure Measures According to Nonactive Travel Mode (n = 44 988)

	Only public transporter	Only car/motor	Public transporter+ car/motor
White matter hyperintensity (mm^3)	0 (ref.)	-0.033 (-0.082, 0.017)	0.035 (-0.031, 0.102)
Gray matter volume (mm^3)	0 (ref.)	0.020 (-0.022, 0.063)	0.012 (-0.046, 0.069)
White matter volume (mm^3)	0 (ref.)	-0.007 (-0.061, 0.048)	0.015 (-0.059, 0.088)
Peripheral cortical gray matter volume (mm^3)	0 (ref.)	0.028 (-0.015, 0.072)	-0.002 (-0.061, 0.057)
Ventricular cerebrospinal fluid volume (mm^3)	0 (ref.)	**-0.079 (-0.128, -0.030)**	-0.064 (-0.130, 0.002)
Hippocampal volume (mm^3)	0 (ref.)	0.011 (-0.043, 0.065)	0.028 (-0.044, 0.100)
Subcortical volumes (mm^3)	0 (ref.)	0.000 (-0.048, 0.048)	0.013 (-0.052, 0.077)
Total volume (mm^3)	0 (ref.)	0.010 (-0.037, 0.056)	0.016 (-0.047, 0.078)

Note: Z-standardized mean differences were calculated by linear regression models adjusted for age, sex, race, education, Townsend deprivation index, assessment centers, alcohol consumption, smoking status, BMI, IPAQ activity group, employment status, diabetes, dyslipidemia, hypertension, CVD, depression, long-standing illness, disability or infirmity, cognitive function (i.e., reaction time), and genetic variables (APOE ε4 carrier status). The volume of white matter hyperintensity was log-transformed before z-standardization because of skew distribution.

© 2025 Hou C et al. *JAMA Network Open*.

eTable 17. Subgroup Analysis of the Association Between Travel Mode and the Risk of All-Cause Dementia, YOD, LOD, and AD by Genetic Risk

	APOE ε4 carriers	N	Non-active	Walking	Mixed-walking	Cycling and mixed-cycling	*P* for interaction
All-cause dementia	No	343335	1.00 (ref.)	0.99 (0.87, 1.12)	0.93 (0.87, 1.00)	0.74 (0.63, 0.87)	**0.0231**
	Yes	136388	1.00 (ref.)	1.14 (1.02, 1.28)	0.94 (0.88, 1.00)	0.88 (0.76, 1.02)	
YOD	No	194008	1.00 (ref.)	0.96 (0.64, 1.45)	1.03 (0.81, 1.32)	0.69 (0.39, 1.21)	0.7631
	Yes	271690	1.00 (ref.)	1.21 (0.74, 1.97)	0.88 (0.63, 1.21)	0.48 (0.22, 1.06)	
LOD	No	240322	1.00 (ref.)	0.99 (0.87, 1.13)	0.93 (0.87, 1.00)	0.75 (0.63, 0.89)	**0.0363**
	Yes	94617	1.00 (ref.)	1.13 (1.01, 1.27)	0.94 (0.88, 1.00)	0.91 (0.78, 1.05)	
AD	No	343335	1.00 (ref.)	1.04 (0.85, 1.28)	0.95 (0.85, 1.06)	0.68 (0.51, 0.90)	0.1230
	Yes	136388	1.00 (ref.)	1.20 (1.03, 1.39)	0.95 (0.87, 1.03)	0.84 (0.69, 1.03)	

Adjusted for age, sex, race, education, Townsend deprivation index, assessment centers, alcohol consumption, smoking status, BMI, IPAQ activity group, employment status, diabetes, dyslipidemia, hypertension, CVD, depression, long-standing illness, disability or infirmity, cognitive function (i.e., reaction time).

© 2025 Hou C et al. *JAMA Network Open*.

eTable 18. Stratified Analysis for the Association Between Travel Mode and Incidence of All-Cause Dementia, YOD, LOD, and AD

		N	Non-active	Walking	Mixed-walking	Cycling and mixed-cycling	*P* for interaction
All-cause dementia							
Sex	Male	218993	1.00 (ref.)	1.15 (1.02, 1.29)	0.94 (0.88, 1.01)	0.84 (0.74, 0.95)	0.5860
	Female	260730	1.00 (ref.)	0.99 (0.88, 1.13)	0.94 (0.88, 1.00)	0.76 (0.62, 0.92)	
Townsend deprivation index	Above median value (high deprivation)	239582	1.00 (ref.)	1.01 (0.91, 1.13)	0.90 (0.84, 0.96)	0.79 (0.68, 0.92)	0.0579
	Below median value (low deprivation)	239552	1.00 (ref.)	1.18 (1.03, 1.34)	0.98 (0.91, 1.05)	0.84 (0.72, 0.97)	
YOD							
Sex	Male	120700	1.00 (ref.)	1.05 (0.68, 1.63)	1.05 (0.80, 1.38)	0.65 (0.38, 1.13)	0.4737
	Female	150990	1.00 (ref.)	1.03 (0.66, 1.62)	0.87 (0.66, 1.15)	0.54 (0.24, 1.25)	
Townsend deprivation index	Above median value (high deprivation)	135659	1.00 (ref.)	1.07 (0.75, 1.54)	0.88 (0.68, 1.13)	0.62 (0.34, 1.10)	0.2359
	Below median value (low deprivation)	135619	1.00 (ref.)	0.93 (0.48, 1.79)	1.12 (0.82, 1.52)	0.59 (0.28, 1.23)	
LOD							
Sex	Male	153418	1.00 (ref.)	1.16 (1.03, 1.31)	0.93 (0.87, 1.00)	0.86 (0.75, 0.98)	0.8352
	Female	181521	1.00 (ref.)	0.98 (0.86, 1.12)	0.95 (0.88, 1.01)	0.78 (0.63, 0.96)	
Townsend deprivation index	Above median value (high deprivation)	167312	1.00 (ref.)	1.00 (0.89, 1.12)	0.90 (0.84, 0.96)	0.82 (0.71, 0.95)	0.1297
	Below median value (low deprivation)	167291	1.00 (ref.)	1.21 (1.05, 1.39)	0.97 (0.91, 1.05)	0.85 (0.72, 0.99)	
AD							
Sex	Male	218993	1.00 (ref.)	1.28 (1.08, 1.51)	0.95 (0.86, 1.05)	0.77 (0.63, 0.94)	0.8229
	Female	260730	1.00 (ref.)	1.02 (0.85, 1.22)	0.95 (0.87, 1.04)	0.80 (0.60, 1.07)	
	Above median value (high deprivation)	239582	1.00 (ref.)	1.02 (0.87, 1.21)	0.92 (0.83, 1.01)	0.72 (0.57, 0.92)	0.3535

© 2025 Hou C et al. *JAMA Network Open*.

Townsend deprivation index	Below median value (low deprivation)	239552	1.00 (ref.)	1.33 (1.10, 1.60)	0.97 (0.88, 1.07)	0.83 (0.66, 1.05)	

Adjusted for age, sex, race, education, Townsend deprivation index, assessment centers, alcohol consumption, smoking status, BMI, IPAQ activity group, employment status, diabetes, dyslipidemia, hypertension, CVD, depression, long-standing illness, disability or infirmity, cognitive function (i.e., reaction time), and genetic variables (APOE ε4 carrier status).

© 2025 Hou C et al. *JAMA Network Open*.

eTable 19. The Association of Travel Mode With Incident Dementia Without Adjusting for the IPAQ

	Non-active	Walking	Mixed-walking	Cycling and mixed-cycling
all-cause dementia	1.00 (ref.)	1.05 (0.97 ,1.14)	**0.93 (0.89 ,0.97)**	**0.79 (0.71 ,0.88)**
AD	1.01 (ref.)	**1.14 (1.01 ,1.28)**	0.95 (0.88 ,1.01)	**0.78 (0.66 ,0.92)**
LOD	1.00 (ref.)	1.05 (0.96 ,1.15)	**0.93 (0.88 ,0.97)**	**0.81 (0.73 ,0.90)**
YOD	1.00 (ref.)	1.02 (0.75 ,1.40)	0.95 (0.78 ,1.15)	**0.58 (0.37 ,0.91)**

Adjusted for age, sex, race, education, Townsend deprivation index, assessment centers, alcohol consumption, smoking status, BMI, diabetes, dyslipidemia, hypertension, CVD, depression, long-standing illness, disability or infirmity, cognitive function (i.e., reaction time), and genetic variables (APOE ε4 carrier status).

© 2025 Hou C et al. *JAMA Network Open*.

eTable 20. The Association of Travel Mode With Incident Dementia Without the Aged <60 Years' Inclusion Criterion for YOD

	Non-active	Walking	Mixed-walking	Cycling and mixed-cycling
YOD	1.00 (ref.)	1.11 (0.82 ,1.50)	0.92 (0.76 ,1.11)	**0.54 (0.35 ,0.86)**
	only public transporter	only car/motor	public transporter+ car/motor	
YOD	1.00 (ref.)	0.77 (0.57 ,1.04)	0.68 (0.40 ,1.15)	

Adjusted for age, sex, race, education, Townsend deprivation index, assessment centers, alcohol consumption, smoking status, BMI, IPAQ activity group, diabetes, dyslipidemia, hypertension, CVD, depression, long-standing illness, disability or infirmity, cognitive function (i.e., reaction time), and genetic variables (APOE ε4 carrier status).

© 2025 Hou C et al. *JAMA Network Open*.

eTable 21. The Association of Commuting Mode or Travel Mode With Incident Dementia in Employed Participants

	Non-active	Walking	Mixed-walking	Cycling and mixed-cycling
**Commuting mode**				
All-cause dementia	1.00 (ref.)	1.08 (0.88, 1.32)	0.89 (0.73, 1.08)	0.80 (0.63, 1.02)
AD	1.00 (ref.)	1.07 (0.79, 1.44)	0.94 (0.70, 1.25)	**0.63 (0.41, 0.96)**
LOD	1.00 (ref.)	1.09 (0.88, 1.36)	0.87 (0.70, 1.08)	0.85 (0.65, 1.10)
YOD	1.00 (ref.)	0.94 (0.55, 1.59)	0.95 (0.62, 1.45)	0.70 (0.39, 1.24)
**Travel mode**				
All-cause dementia	1.00 (ref.)	1.10 (0.93, 1.32)	0.92 (0.83, 1.02)	0.85 (0.69, 1.04)
AD	1.00 (ref.)	1.19 (0.91, 1.54)	0.90 (0.77, 1.06)	0.75 (0.54, 1.05)
LOD	1.00 (ref.)	1.14 (0.94, 1.38)	0.90 (0.81, 1.01)	0.93 (0.75, 1.16)
YOD	1.00 (ref.)	0.87 (0.55, 1.39)	0.93 (0.72, 1.19)	**0.49 (0.27, 0.89)**
**Travel mode (model + commuting)**				
All-cause dementia	1.00 (ref.)	1.11 (0.91, 1.34)	0.91 (0.81, 1.01)	0.87 (0.67, 1.11)
AD	1.00 (ref.)	1.27 (0.96, 1.68)	0.90 (0.75, 1.07)	0.87 (0.59, 1.30)
LOD	1.00 (ref.)	1.15 (0.93, 1.41)	0.90 (0.79, 1.01)	0.97 (0.74, 1.27)
YOD	1.00 (ref.)	0.87 (0.52, 1.43)	0.90 (0.69, 1.18)	**0.42 (0.20, 0.88)**

Adjusted for age, sex, race, education, Townsend deprivation index, assessment centers, alcohol consumption, smoking status, BMI, IPAQ activity group, diabetes, dyslipidemia, hypertension, CVD, depression, long-standing illness, disability or infirmity, cognitive function (i.e., reaction time), and genetic variables (APOE ε4 carrier status).

© 2025 Hou C et al. *JAMA Network Open*.

eTable 22. Association Between Travel Mode and Incident All-Cause Dementia, AD, YOD, and LOD by Complete Cases

	Cases/N	Non-active	Walking	Mixed-walking	Cycling and mixed-cycling
**all-cause dementia**	6583/384872	1.00 (ref.)	1.06 (0.96, 1.17)	**0.93 (0.88, 0.98)**	**0.82 (0.73, 0.92)**
**AD**	2929/384872	1.00 (ref.)	**1.15 (1.02, 1.31)**	0.96 (0.90, 1.03)	**0.80 (0.68, 0.94)**
**YOD**	389/222309	1.00 (ref.)	1.09 (0.76, 1.56)	0.94 (0.75, 1.18)	0.60 (0.36, 1.00)
**LOD**	6166/264796	1.00 (ref.)	1.05 (0.95, 1.17)	**0.93 (0.88, 0.98)**	**0.84 (0.75, 0.95)**

Adjusted for age, sex, race, education, Townsend deprivation index, assessment centers, alcohol consumption, smoking status, BMI, IPAQ activity group, employment status, diabetes, dyslipidemia, hypertension, CVD, depression, long-standing illness, disability or infirmity, cognitive function (i.e., reaction time), and genetic variables (APOE ε4 carrier status).

© 2025 Hou C et al. *JAMA Network Open*.

eTable 23. Association Between Travel Mode and Incident All-Cause Dementia, AD, YOD, and LOD Using Fine and Gray Models for Competing Risk

	Non-active	Walking	Mixed-walking	cycling and mixed-cycling
all-cause dementia	1.00 (ref.)	1.09 (1.00, 1.18)	**0.95 (0.91, 0.99)**	**0.83 (0.75, 0.93)**
AD	1.00 (ref.)	1.15 (1.02, 1.31)	0.96 (0.90, 1.03)	**0.80 (0.68, 0.94)**
YOD	1.00 (ref.)	1.05 (0.77, 1.44)	0.97 (0.80, 1.17)	**0.61 (0.38, 0.96)**
LOD	1.00 (ref.)	1.09 (0.99, 1.19)	**0.95 (0.90, 1.00)**	**0.85 (0.77, 0.95)**

Adjusted for age, sex, race, education, Townsend deprivation index, assessment centers, alcohol consumption, smoking status, BMI, IPAQ activity group, employment status, diabetes, dyslipidemia, hypertension, CVD, depression, long-standing illness, disability or infirmity, cognitive function (i.e., reaction time), and genetic variables (APOE ε4 carrier status).

© 2025 Hou C et al. *JAMA Network Open*.
