# Supplementary material for: Active Travel Mode and Incident Dementia and Brain Structure
Source: JAMA Netw Open. 2025 Jun 9;8(6):e2514316. doi: 10.1001/jamanetworkopen.2025.14316 (PMC12150188; doi:10.1001/jamanetworkopen.2025.14316)
Supplement: Supplement 2. — Data Sharing Statement [file jamanetwopen-e2514316-s002.pdf]

Data Sharing Statement

Hou. Active Travel Mode and Incident Dementia and Brain Structure. *JAMA Netw Open*. Published June 09, 2025. doi:10.1001/jamanetworkopen.2025.14316

Data
Data available: No

Additional Information
Explanation for why data not available: This data requires application to the UK Biobank for use.
